# A Novel Computer-Based Set-Up to Study Movement Coordination in Human Ensembles

**DOI:** 10.3389/fpsyg.2017.00967

**Published:** 2017-06-09

**Authors:** Francesco Alderisio, Maria Lombardi, Gianfranco Fiore, Mario di Bernardo

**Affiliations:** ^1^Department of Engineering Mathematics, University of BristolBristol, United Kingdom; ^2^Department of Electrical Engineering and Information Technology, University of Naples Federico IINaples, Italy

**Keywords:** multiplayer games, social interaction, human ensembles, coordination, group synchronization, human-robot interaction, computer software, client-server

## Abstract

Existing experimental works on movement coordination in human ensembles mostly investigate situations where each subject is connected to all the others through direct visual and auditory coupling, so that unavoidable social interaction affects their coordination level. Here, we present a novel computer-based set-up to study movement coordination in human groups so as to minimize the influence of social interaction among participants and implement different visual pairings between them. In so doing, players can only take into consideration the motion of a designated subset of the others. This allows the evaluation of the exclusive effects on coordination of the structure of interconnections among the players in the group and their own dynamics. In addition, our set-up enables the deployment of virtual computer players to investigate dyadic interaction between a human and a virtual agent, as well as group synchronization in mixed teams of human and virtual agents. We show how this novel set-up can be employed to study coordination both in dyads and in groups over different structures of interconnections, in the presence as well as in the absence of virtual agents acting as followers or leaders. Finally, in order to illustrate the capabilities of the architecture, we describe some preliminary results. The platform is available to any researcher who wishes to unfold the mechanisms underlying group synchronization in human ensembles and shed light on its socio-psychological aspects.

## 1. Introduction

Interpersonal coordination between the motion of two individuals performing a joint task has been extensively studied over the past few decades (Schmidt and Turvey, 1994; Richardson et al., 2007; Oullier et al., 2008; Schmidt and Richardson, 2008; Marsh et al., 2009; Varlet et al., 2011; Walton et al., 2015; Słowiński et al., 2016); a recent example being that of the *mirror game*, presented as paradigmatic case of study where human participants (HP) imitate each other's movements in a pair (Noy et al., 2011). In general, multiplayer scenarios have been investigated less than those involving only two participants, because of practical problems in running the experiments and the lack of models accounting for movement coordination in human groups, in contrast to the numerous studies dealing with animal groups (Couzin et al., 2005; Nagy et al., 2010, 2013; Zienkiewicz et al., 2015).

In this methodological paper we present a novel computer-based set-up that we named “Chronos” (a tool to study synCHRONizatiOn and coordination in human ensembleS), which allows participants to perform a joint task from a distance, both in dyads and in groups. The platform extends the mirror game to multiplayer scenarios, allowing each subject in the group to run a serious computer game where s/he can move the position of an object on her/his computer screen and see traces of the objects moved by the other players. The software makes it possible to show on each screen only the traces of a designated subset of the players in the group (as decided by an Administrator), so that different interaction patterns among its members can be implemented. To prevent any form of social interaction when in the same environment, participants are visually separated by barriers and hear white noise (through headphones connected to their computers) isolating them from any external sound. Alternatively, players can join the group remotely. Therefore, subjects have no information on the identity of those they are interacting with and receive no direct visual or behavioral cues from other members in the group.

Previous existing results on multiplayer human coordination include studies on rocking chairs (Frank and Richardson, 2010; Richardson et al., 2012; Alderisio et al., 2016b), group synchronization of arm movements and respiratory rhythms (Codrons et al., 2014), music (Glowinski et al., 2013; Badino et al., 2014; Volpe et al., 2016), and sport activities (Wing and Woodburn, 1995; Yokoyama and Yamamoto, 2011). However, in these papers the features and the level of coordination are not explicitly correlated to the way the players interact (i.e., the structure of their connections), as all the subjects involved share direct visual and auditory coupling with all the others, and no other patterns are considered. Moreover, inevitable social interaction affects the level of coordination in the group (Healey et al., 2005; Kauffeld and Meyers, 2009; Passos et al., 2011; D'Ausilio et al., 2012; Duarte et al., 2012, 2013; Glowinski et al., 2013; Cardillo et al., 2014). Indeed, body movements, friendship relationships, shared feelings, particular affinities, and levels of hierarchy have a significant impact on how each individual in the ensemble chooses her/his preferred partner(s) to interact the most with (Baumeister and Leary, 1995; Mäs et al., 2010; Stark et al., 2013). By making it possible to implement different visual pairings and minimize social interactions, our computer-based architecture allows instead to assess the impact on the group coordination level of solely varying the structure of interconnections among its members, a phenomenon that has been suggested to be crucial in determining the level of coordination arising in a human group (Passos et al., 2011; Duarte et al., 2012, 2013; Alderisio et al., 2016c).

In addition, Chronos allows the trace of some objects on the players' screen to be moved by virtual players (VP) driven by a computational cognitive architecture which is described in the rest of the paper. Simulated agents make it possible to further analyze the mechanisms underlying human coordination, explore features that are not easily accessible in ordinary human interactions, and point out interesting aspects of the task that are not immediately obvious from experimental observations (Di Paolo et al., 2008; Froese and Di Paolo, 2010). In so doing, we take inspiration from the *human dynamic clamp* (HDC) originally introduced in Dumas et al. (2014) as a paradigm to control the interactions between a human and a surrogate (virtual agent). By changing the mathematical description of the latter and appropriately tuning its parameters, it is possible to explore different behaviors and tasks, as well as test hypotheses and shed light on how humans interact. In our work we extend the HDC to a multiplayer scenario, thus allowing virtual agents to interact within a group of people.

To illustrate and validate the use of the set-up, we present preliminary experimental results obtained in a group of 5 individuals performing a joint oscillatory task. Specifically, we use the platform to vary the interaction patterns between players and observe the effects of such variations on the coordination level. Rather than being exhaustive, the experiments are reported here to illustrate the capabilities of the new methodology implemented via the platform. Therefore, we leave to future publications a more thorough experimental confirmation of the preliminary findings reported here for illustrative purposes. We also demonstrate that the platform allows the deployment of virtual computer players driven by feedback control algorithms. Specifically, we run preliminary experiments where a VP is enabled to interact with either one or a group of 4 human players, showing that its presence has an effect on their coordination level. Again, these experiments are not meant to be exhaustive but are only included to demonstrate the ability of the novel set-up to run trials involving multiple virtual players (a feature that has not been presented anywhere else in other methods in the literature on movement coordination in groups).

The new platform we present is available for download from https://dibernardogroup.github.io/Chronos to every interested reader.

## 2. Chronos architecture

The proposed computer-based platform is a hardware/software set-up consisting of input/output devices, a centralized unit (server and client-adiministrator) processing data, broadcasting movement information to the various client-players and implementing virtual agents, and a Wi-Fi apparatus connecting all the components together. The central server unit receives position data from the client-players and broadcasts to each position data from a subset of the others, according to the desired structure of interconnections being implemented. For example, in a ring network each client-player will only receive position data from two neighboring client-players. The movements of each human agent are detected by a low-cost position sensor, and individuals interact with each other through their own personal computer, on whose screens the central unit broadcasts the appropriate position trajectories according to the assigned topology (visual interaction patterns). The central unit is also responsible for data management and storage.

**Figure 1:**
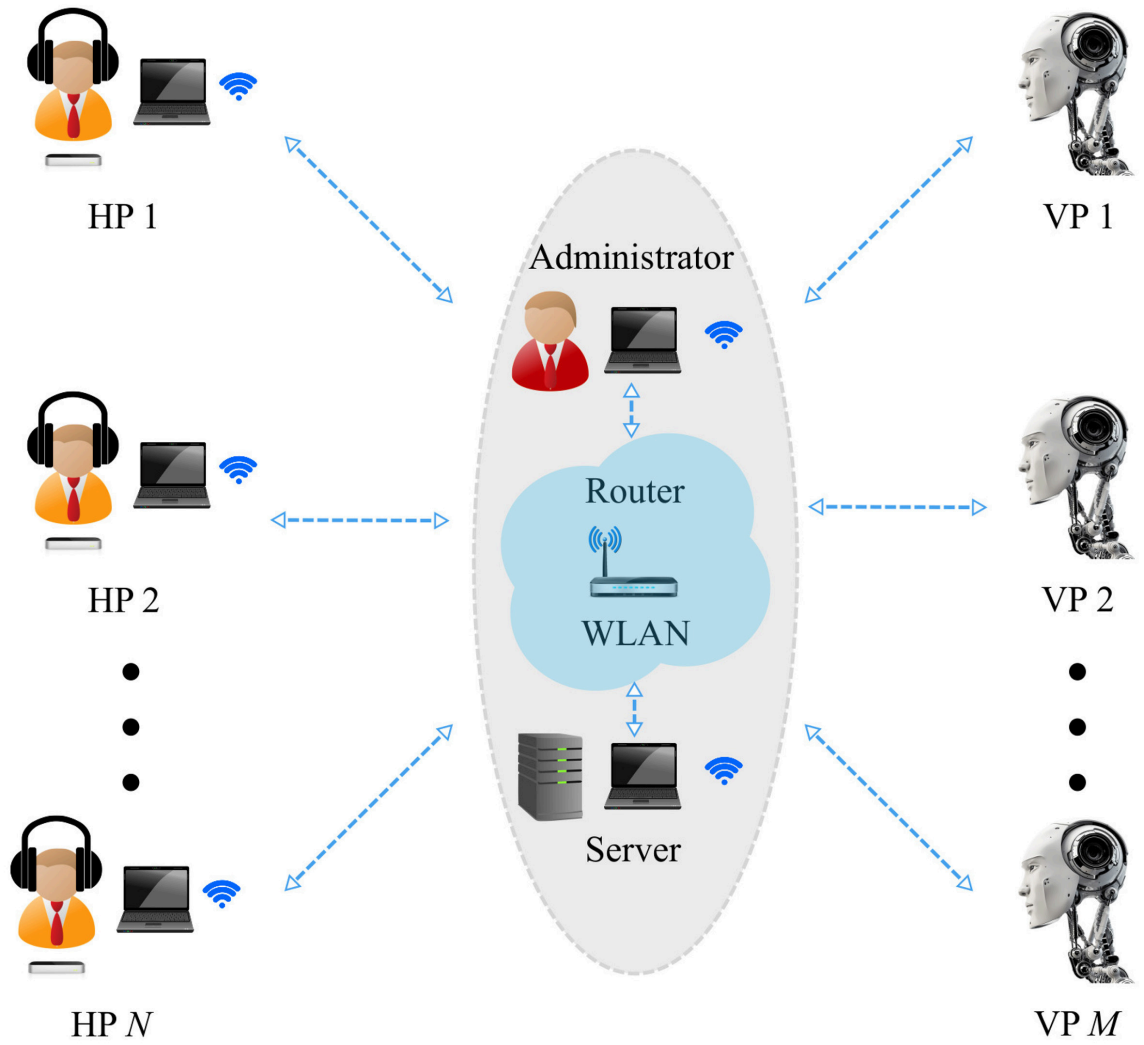
Computer-based set-up architecture. *N* player modules, respectively accessible by one human participant (HP) each, send the Server requests to perform either *Dyadic* or *Group interaction* trials, so that data can be appropriately stored and players can interact with each other in real-time. *N* human players, hearing white noise through headphone sets, move their preferred hand over their own position sensor. They see their own 1D position trajectory and that of the others they are possibly interacting with on their respective computer screen. An Administrator module allows to record (and store through the Server in an appropriate database) the motor signature of a given human participant in *Solo experiments*, and to set the topology of interconnections, the duration of each trial and the model of *M* possible virtual players (VP) in the case of *Dyadic* and *Group interaction*. A Server module implements the virtual players as computerized versions of the human motion, without the need for additional machines or physical entities/robots. All the machines are connected onto the same wireless local area network (WLAN) by means of a dedicated Wi-Fi router.

The proposed set-up is shown in details in Figure 1 for the case of *N* human participants and *M* virtual agents, and described below in all its components (for more information on how to use the set-up and for download of the software, see https://dibernardogroup.github.io/Chronos and Section 1 of Supplementary Material).

### 2.1. Hardware equipment

The hardware equipment consists of:

- *N position sensors*. Each player waves the index finger of her/his preferred hand over a Leap Motion controller (Leap Motion, Inc.) which captures its movements over time as a monodimensional trajectory (Guna et al., 2014); alternatively, a mouse or trackpad can be used.
- *N* + 1 *Personal Computers*. Each position sensor is connected to a PC, such that the recorded position trajectory can be stored after any trial. Participants are able to see their motion and that of the others they are possibly interacting with on their respective computer screens, by means of moving color-coded circles. For each participant, a blue circle represents her/his own motion, whereas orange circles represent those of the others, respectively (see Figure S10). The two coordinates of each circle on the screen are updated according to the position detected by the position sensor: one of them is kept fixed, while the other corresponds to the input received by the sensor. One additional computer is needed to run the server and a GUI that allows the administrator to set the experimental parameters and the desired visual interaction patterns (see Section 2.2). No further machines are needed to implement the *M* virtual players, as the cognitive architecture driving their motion is run by the central server that dispatches their position data to the various clients as required.
- *N headphone sets*. Each player wears headphones through which white noise is transmitted to eliminate possible auditory couplings with the others.
- 1 *router*. It provides Wi-Fi signal in order to allow clients (administrator and players) and server to be logged onto the same wireless local area network (WLAN) through TCP/IP protocol (Forouzan, 2002).

**Figure 2:**
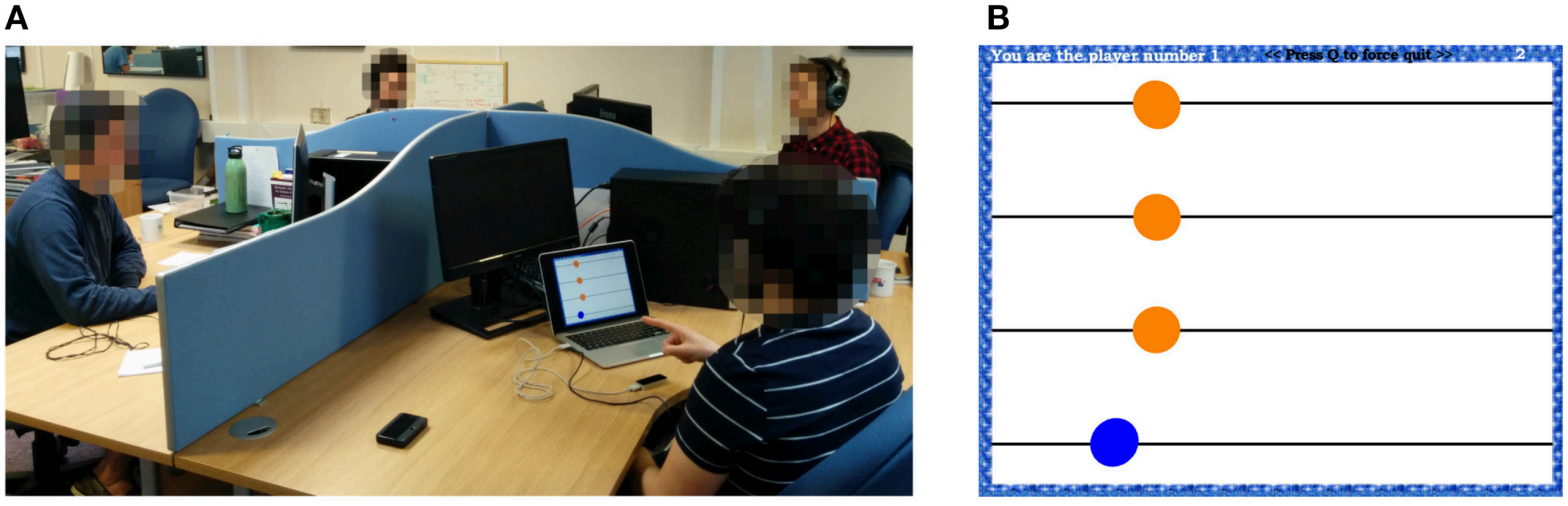
Group interaction experiments. **(A)**: Experimental set-up. Human participants move their preferred hand over a Leap Motion controller while sitting around a table. They are separated by barriers (no direct visual coupling) and wear headphones (no auditory coupling), so that social interaction is removed. **(B)**: User interface. For each participant, a blue circle on the screen represents her/his own motion, while orange circles represent those of the others s/he is possibly coupled with.

Furthermore, barriers are employed to separate the players and prevent them from being directly visually coupled (Figure 2).

### 2.2. Software architecture

The software architecture, which is based on a client-server model (Berson, 1996), consists of:

- *N Player modules*. These modules, respectively accessible by one human player each, provide a user-friendly interface through which participants interact. They send the server requests to perform either *Dyadic* or *Group interaction* trials (see Section 2.4), such that data can be appropriately stored and the trial correctly started.
- 1 *Administrator module*. This module, accessible by the administrator only, carries out different tasks according to the type of experiments being performed (see Section 2.4) through a user-friendly interface. In the case of *Solo experiments*, it allows to record *the individual motor signature* of a given human participant (Słowiński et al., 2016). In the case of *Dyadic interaction*, it allows to set the duration of each trial, the roles played by the two agents, as well as model and parameter of the possible VP to be used in human-virtual player experiments. In the case of *Group interaction*, it allows to set the topology of interactions among the HPs and the duration of each trial, as well as to choose the number of VPs and their models and parameters to be used in mixed human-virtual player experiments.
- 1 *Server module*. It handles communication among different players' machines and connects them onto the WLAN provided by the router. In the case of *Solo experiments*, it deals with the motor signature storage in an appropriate database. In the case of *Dyadic* and *Group interaction*, it manages requests coming from the players so that all the participants can interact in real-time, dealing with trajectories broadcasting (each player sees her/his motion and that of the others s/he is interacting with) and storage.

### 2.3. Virtual player implementation

Let *x*(*t*) ∈ ℝ be the state variable representing the position of the virtual player at time *t*. The system describing its behavior is given by the following dynamical system:

(1) $$\ddot{x}(t)=f(x(t),\dot{x}(t))+u(t)$$

where *f* represents the vector field modeling the inner dynamics of the VP when disconnected from any other agent, ẋ and ẍ represent velocity and acceleration of the VP, and *u* is the control signal modeling how the VP interacts with other players, i.e., its coupling function.

Chronos allows to select different combinations of inner dynamics models and control signals to describe the motion of the virtual player. In what follows, different alternatives are proposed for the VP to exhibit human-like motion features when interacting with one or more partners (Alderisio et al., 2016a,b). Further details on why each of these mathematical models successfully enable a VP to behave in a human-like manner can be found in Zhai et al. (2014a,b, 2015, 2016, 2017).

#### 2.3.1. Inner dynamics models

The alternative models describing the inner dynamics *f* of the virtual player can be listed as follows.

- *Harmonic oscillator*, a linear system given by
  (2) $$f(x,\dot{x})=-(a\dot{x}+bx)$$
  where *a* and *b* represent viscous damping coefficient and the elastic coefficient, respectively.
- *HKB equation*, a nonlinear oscillator given by
  (3) $$f(x,\dot{x})=-(\alpha x^{2}+\beta {\dot{x}}^{2}-\gamma )\dot{x}-\omega^{2}x$$
  where α, β, γ characterize the damping coefficient, while ω is related to the oscillation frequency, respectively.

Chronos gives also the opportunity to describe the behavior of the VP as a *double integrator*, that is a system without any inner dynamics (*f* = 0) whose motion is entirely determined by the coupling with the other agents via the control input *u*(*t*). In this case the system describing the behavior of the VP becomes:

(4) $$\ddot{x}(t)=u(t)$$

#### 2.3.2. Control signal

The different options for the control signal *u* describing the coupling of the virtual player with other agents can be listed as follows.

- *PD control*, a linear control law given by
  (5) $$u=K_{p}(y-x)+K_{\sigma }(\dot{\sigma }-\dot{x})$$
  where *y* is the position of the other agent coupled to the VP, $\dot{\sigma }$ is its reference motor signature as defined in Słowiński et al. (2016), that is a velocity trajectory characterizing some desired human-like kinematic features to be assigned to the VP, and *K*_*p*_ and *K*_σ_ are two control gains. According to the values of *K*_*p*_ and *K*_σ_, the VP acts as a leader (more weight given to *K*_σ_ so that the VP priority is to minimize the mismatch between its own velocity and that of the prerecorded motor signature) or as a follower (more weight given to *K*_*p*_ and hence higher priority to reducing the mismatch between the VP position and that of the other player).
- *Adaptive control*, a nonlinear control law which changes according to the nature of the experiment being carried out.
  - When the VP acts as a follower, it is given by
    (6) $$u=[\psi +\chi {\left(x-y\right)}^{2}](\dot{x}-\dot{y})-Ce^{-\delta {\left(\dot{x}-\dot{y}\right)}^{2}}(x-y)$$
    with
    (7) $$\dot{\psi }=-\frac{1}{\psi }[(x-y)(\dot{x}-\dot{y})+{\left(x-y\right)}^{2}]$$
    (8) $$\dot{\chi }=-\frac{1}{\chi }(\dot{x}-\dot{y})[f(x,\dot{x})+u]$$
    where *y* and ẏ are position and velocity of the other agent coupled to the VP, *C* and δ are control parameters, and ψ and χ are adaptive parameters. Note that in this case no motor signature can be assigned to the VP.
  - When the VP acts as a leader, the control input is set as
    (9) $$\begin{array}{l} u=\lambda \left(\left[\psi +\chi {\left(x-\sigma \right)}^{2}](\dot{x}-\dot{\sigma })-Ce^{-\delta {\left(\dot{x}-\dot{\sigma }\right)}^{2}}(x-\sigma \right)\right)\\ \;+\;(1-\lambda )K(y-x) \end{array}$$
    where λ: = *e*^−δ|*x*−*y*|^, *K* is a control parameter, σ and $\dot{\sigma }$ are desired position and velocity profiles (motor signature) that allow the VP to generate spontaneous motion, and all the other quantities have been previously defined.

Note that when the VP is influenced by the motion of two or more agents, as it might happen in the *Group interaction* trials (see Section 2.4), then *y* and ẏ are appropriately replaced by average position and velocity of all the agents connected to the VP, respectively.

### 2.4. Types of possible experiments that can be run

**Figure 3:**
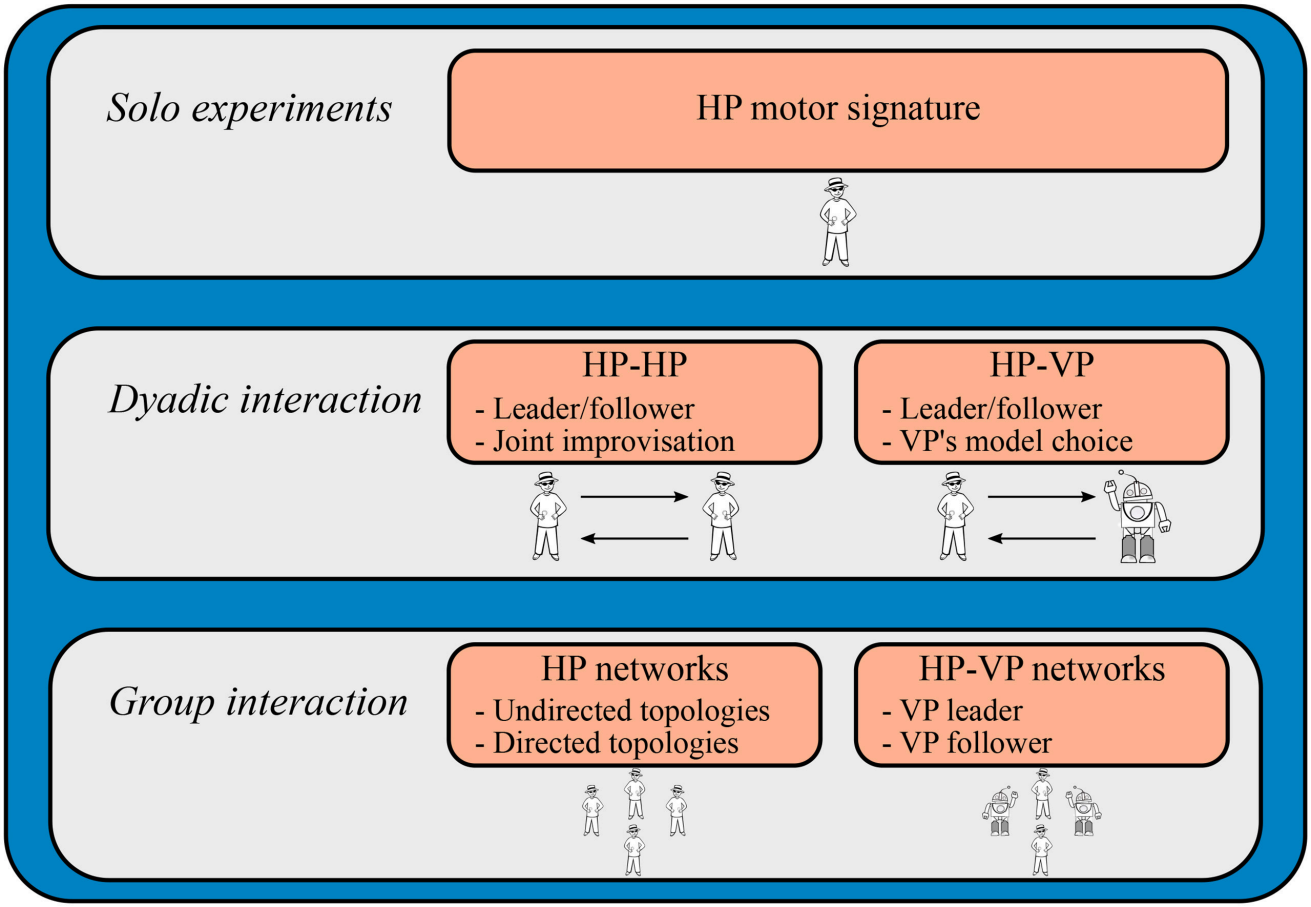
Choice of experiments through our proposed technology. *Solo experiments*: participants are separately asked to generate some spontaneous movement of their preferred hand in isolation, so that their individual motor signature can be recorded. *Dyadic interaction*: both HP-HP (two human participants can either interact in a Leader-Follower or in a Joint improvisation condition) and HP-VP trials (a human participant is asked to either lead or follow a virtual agent, whose mathematical description for its dynamics can be chosen among different models) can be performed. *Group interaction*: any kind of structure of interconnections can be set among the players. Human participants are asked to synchronize the motion of their preferred hand with that of the others they are topologically connected to (HP networks), with the possibility of implementing virtual agents in the group (HP-VP networks), which can be set to act either as follower or leader.

All types of experiments that can be performed through the proposed technology are listed below and summarized in Figure 3.

1. *Solo experiments*. These experiments involve only one agent at a time. Participants are separately asked to generate some spontaneous movement of their preferred hand, so that their individual motor signature as defined in Słowiński et al. (2016) can be acquired.
2. *Dyadic interaction*. These experiments involve only two agents. Two kinds of trials can be performed:
  - HP-HP trials: human participants can either interact in a Leader-Follower condition (one of them leads the game and the other tracks her/his hand movements), or in a Joint Improvisation condition (there is no designation of leader and follower, the two participants are asked to create an interesting and synchronized motion of their preferred hands).
  - HP-VP trials: a human participant is asked to either lead or follow a virtual agent, whose mathematical description for its dynamics can be chosen among different models (see Section 2.3).
3. *Group interaction*. These experiments involve two or more agents, where any kind of structure of interconnections among them can be set. In particular, the network topology can be either *undirected* (participant *i* sees the motion of participant *j* if and only if participant *j* sees the motion of participant *i*) or *directed* (the previous condition is not verified). Two kinds of networks can be implemented:
  - HP networks: human participants are asked to synchronize the motion of their preferred hand with that of the others they are topologically connected with.
  - mixed HP-VP networks: one or more participants of the group are virtual agents, which can be set to act either as followers or leaders, according to how much attention they pay to tracking the motion of the other group members they are connected with or generating spontaneous movements, respectively (see Section 2.3).

Note that, differently from *Dyadic interaction*, in the case of *Group interaction* trials between only two agents it is possible to assign directions to the coupling between players, as well as perform *in-silico* trials between two virtual players.

## 3. Application

### 3.1. Participants

A total of 9 people participated in the experiments: 1 female and 8 males (all the participants were right handed, and none of them had physical and mental illnesses or disabilities). The participants, who volunteered to take part in the experiments, were master students, Ph.D. students, and Postdoctoral Research Associates from the University of Bristol. The experiments took place in three separate sessions.

This study was reviewed and approved by the Ethics Office of the University of Bristol. All subjects gave written informed consent in accordance with the Declaration of Helsinki. Ethical harm was minimized: due care was taken to avoid coercion or exploitation, protect confidentiality, minimize the risk of physical and psychological harm and respect autonomy. Any information obtained in connection with this study remained confidential, and participants' identity is kept anonymous.

### 3.2. Synchronization metrics

Before describing in details some representative study cases illustrating the features and capabilities of Chronos, we report the metrics used in this work to assess players' performance in *Dyadic* and *Group interaction* experiments. Note that such metrics are independent of the architecture we propose. Indeed, depending on the hypotheses a researcher is interested in investigating, other metrics can be employed to analyze the data stored through Chronos, as for example those proposed in Di Paolo et al. (2008), Froese and Di Paolo (2010), Snapp-Childs et al. (2011).

Let *x*_*k*_(*t*) ∈ ℝ ∀*t* ∈ [0, *T*] be the continuous time series representing the motion of the *k*th agent's preferred hand, with *k* ∈ {1, 2, …, *N*}, where *N* is the number of individuals and *T* is the duration of the experiment. Let *x*_*k*_[*t*_*i*_] ∈ ℝ, with *k* ∈ {1, 2, …, *N*} and *i* ∈ {1, 2, …, *N*_*T*_}, be the discrete time series of the position of the *k*th agent, obtained after sampling *x*_*k*_(*t*) at time instants *t*_*i*_, where *N*_*T*_ is the number of time steps of duration $\Delta T:=\frac{T}{N_{T}}$, that is the sampling period. Let θ_*k*_(*t*) ∈ [−π, π] be the phase of the *k*th agent, which can be estimated by making use of the Hilbert transform of the signal *x*_*k*_(*t*) as detailed in Kralemann et al. (2008).

In *Dyadic interaction experiments*, the relative phase ϕ_*d*_*h,k*__(*t*): = θ_*h*_(*t*) − θ_*k*_(*t*) ∈ [−π, π] was used to check whether the assigned roles of leader and follower were respected by participants *h* and *k* at time *t*. Indeed, by defining ϕ_*d*_*h,k*__ as the difference between the phase of the leader (player *h*) and that of the follower (player *k*), positive values indicate that the designated leader is effectively leading the game while interacting with the follower (Zhai et al., 2016).

In addition, the symmetric *dyadic synchronization index* ρ_*d*_*h,k*__ ∈ [0, 1] originally introduced in Richardson et al. (2012) and defined as

(10) $$\rho_{d_{h,k}}:=\left|\frac{1}{T}\int_{0}^{T}e^{j\phi_{d_{h,k}}(t)}\;dt\right|≃\left|\frac{1}{N_{T}}\sum_{i=1}^{N_{T}}e^{j\phi_{d_{h,k}}[t_{i}]}\right|$$

was used to quantify the average coordination level between agents *h* and *k* over time: the closer ρ_*d*_*h,k*__ = ρ_*d*_*k,h*__ is to 1, the lower the phase mismatch is between agents *h* and *k* over the whole trial.

The root mean square (RMS) of the normalized position error ϵ_*h,k*_ ∈ [0, 100]% defined as

(11) $$\begin{array}{l} \epsilon_{h,k}\;:=\;\frac{1}{L}\sqrt{\frac{1}{T}\int_{0}^{T}{\left(x_{h}(t)-x_{k}(t)\right)}^{2}\;dt}\\ \;≃\frac{1}{L}\sqrt{\frac{1}{N_{T}}\sum_{i\;=\;1}^{N_{T}}{\left(x_{h}[t_{i}]-x_{k}[t_{i}]\right)}^{2}} \end{array}$$

where *L* refers to the range of admissible position (e.g., the range of motion detected by the Leap Motion controller), was employed as a measure of the position mismatch (expressed in percentage) between the two agents: the lower ϵ_*h,k*_ is, the lower the position mismatch is between agents *h* and *k*.

When *N* > 2 (*Group interaction*), further indices can be used to measure the coordination level of each participant in the group, as well as that of the entire ensemble. Firstly, the *cluster phase* or *Kuramoto order parameter* is defined both in its complex form *q*′(*t*) ∈ ℂ and in its real form *q*(*t*) ∈ [−π, π] as

(12) $$q^{\prime }(t):=\frac{1}{N}\sum_{k\;=\;1}^{N}e^{j\theta_{k}(t)},\;q(t)\;:=\text{atan}2\left(ℑ(q^{\prime }(t)),ℜ(q^{\prime }(t))\right)$$

which can be regarded as the average phase of the group at time *t*. Secondly, denoting with ϕ_*k*_(*t*): = θ_*k*_(*t*) − *q*(*t*) the relative phase between the *k*th participant and the group phase at time *t*, the relative phase between the *k*th participant and the group averaged over the time interval [0, *T*] is defined both in its complex form ${\bar{\phi }}_{k}^{\prime }\in \mathbb{C}$ and in its real form ${\bar{\phi }}_{k}\in \left[-\pi ,\pi \right]$ as

(13) $$\begin{array}{l} {\bar{\phi }}_{k}^{\prime }\;:=\;\frac{1}{T}\int_{0}^{T}e^{j\phi_{k}(t)}\;dt≃\frac{1}{N_{T}}\sum_{i\;=\;1}^{N_{T}}e^{j\phi_{k}[t_{i}]},\\ {\bar{\phi }}_{k}\;:=\text{ atan}2\left(ℑ({{\bar{\phi }}^{\prime }}_{k}),ℜ({{\bar{\phi }}^{\prime }}_{k})\right) \end{array}$$

The *individual synchronization index* ρ_*k*_ ∈ [0, 1] originally introduced in Richardson et al. (2012) and defined as

(14) $$\rho_{k}:=\left|{\bar{\phi }}_{k}^{\prime }\right|$$

was then used to quantify the synchronization level of the *k*th participant over the whole trial duration: the closer ρ_*k*_ is to 1, the smaller the average phase mismatch between agent *k* and the group. Similarly, the *group synchronization index* ρ_*g*_(*t*) ∈ [0, 1] defined as

(15) $$\rho_{g}(t):=\frac{1}{N}\left|\sum_{k\;=\;1}^{N}e^{j\left(\phi_{k}(t)-{\bar{\phi }}_{k}\right)}\right|$$

was used to quantify the synchronization level of the entire group at time *t*: the closer ρ_*g*_(*t*) is to 1, the smaller the average phase mismatch of the agents in the group is at time *t*. The mean synchronzation level of the group ρ_*g*_ ∈ [0, 1] over the total duration of the performance can consequently be estimated as:

(16) $$\rho_{g}:=\frac{1}{T}\int_{0}^{T}\rho_{g}(t)\;dt≃\frac{1}{N_{T}}\sum_{i\;=\;1}^{N_{T}}\rho_{g}[t_{i}]$$

### 3.3. Representative study cases

To better illustrate the features and capabilities of Chronos, we apply the platform to some representative scenarios. Specifically, we consider first the case of a dyadic interaction between two players, we then move to studying group coordination in an ensemble of 5 players with and without the presence of virtual players. Illustrations of the interfaces exhibited in the different scenarios can be found in Figures S2–S9.

All the experiments involved participants sitting around a table and moving the index finger of their preferred hand as smoothly as possible over a Leap Motion controller, along a direction required to be straight and parallel to the floor. The instruction to move smoothly was given to keep the attentional level of the participants as high as possible throughout all the experiments. Data was originally stored with a frequency rate of 10 Hz, and then underwent cubic interpolation (100 Hz, see Figure S1).

Remark 1. *In general, the Leap Motion controller allows to detect the 3D position of both hands by providing several triplets (*x, y, z*) for each of them. In particular,* 4 *triplets are provided for the thumb, whereas* 5 *triplets are provided for the other fingers, in addition to two more triplets representing wrist and palm position, respectively. Given the nature of the task here considered (preferred hand's index-finger 1D motion along the *x*-axis of the sensor), only the *x*-coordinate of the index finger's tip position was recorded for each participant.*

Remark 2. *A too high sampling frequency could cause delays in the communication among different machines. Indeed, regardless of the input device employed as position sensor, increasing the sample rate would lead to a larger quantity of data to be acquired, stored, and then sent to the server from different machines at the same time, and hence to possible undesired communication delays deteriorating the effectiveness of the task. Despite the Leap Motion controller providing a value of sampling frequency up to* 40 *Hz (Guna et al., 2014), we found that* 10 *Hz was low enough to avoid delays, yet sufficiently high to guarantee an adequate number of samples to be analyzed. An upsampling was performed* a posteriori *for the sake of a more accurate analysis.*

Remark 3. *To make sure that the chosen sampling frequency allowed for synchrony among all the machines involved in the experiments, we run a mock group synchronization trial and verified that the position acquired on the least computationally powerful machine was broadcast in real time to all the others. Any undesired delay when broadcasting the position of the human players could lead to additional phase mismatches, thus deteriorating the metrics introduced in Section 3.2. Furthermore, note that VPs do not introduce delays as they are locally implemented on the administrator's machine.*

#### 3.3.1. Solo experiments

Four participants were asked to separately perform 4 trials, each of duration 60 s. Specifically, each participant was told to perform 2 trials while producing a sinusoidal-like wave at their own natural oscillation frequency, and then 2 more trials while producing an interesting non-periodic motion representing their motor signature (Słowiński et al., 2016).

These experiments were carried out in the first session (see Section 1.1 of Supplementary Material for more details on how to perform solo trials via Chronos, and Figure S11 for an example of individual motor signature).

#### 3.3.2. Dyadic interaction experiments

The same four participants were grouped in two pairs: players 1 and 2 formed Dyad 1, while players 3 and 4 formed Dyad 2, respectively.

**Figure 4:**
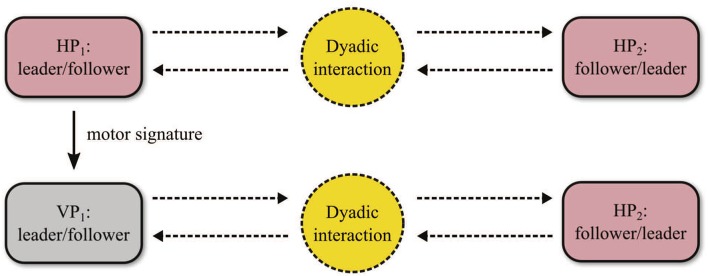
Dyadic interaction experiments. Two human participants are asked to perform trials in a Leader-Follower condition. Then one of them is replaced by a virtual player, which is provided with the same kinematic features (motor signature) as those of the substituted human player. The virtual agent plays the role of the replaced human participant in the HP-HP interaction (Leader or Follower).

- Each player was asked to perform 2 HP-HP trials of duration 30 s in Leader-Follower condition, and did not know the identity of her/his partner. In particular, players 1 and 4 acted as leader, while players 2 and 3 as followers.
- Then, for each pair, either of the two players was replaced by a virtual agent (modeled by HKB equation with PD control) fed with the motor signature, captured during *Solo experiments*, of the human player it was substituting (Figure 4). In particular, players 1 and 3 were replaced, and players 2 and 4 were not informed on this (they believed they were still interacting with their human partner). Once again, 2 HP-VP trials of duration 30s were performed for each pair in Leader-Follower condition.

**Figure 5:**
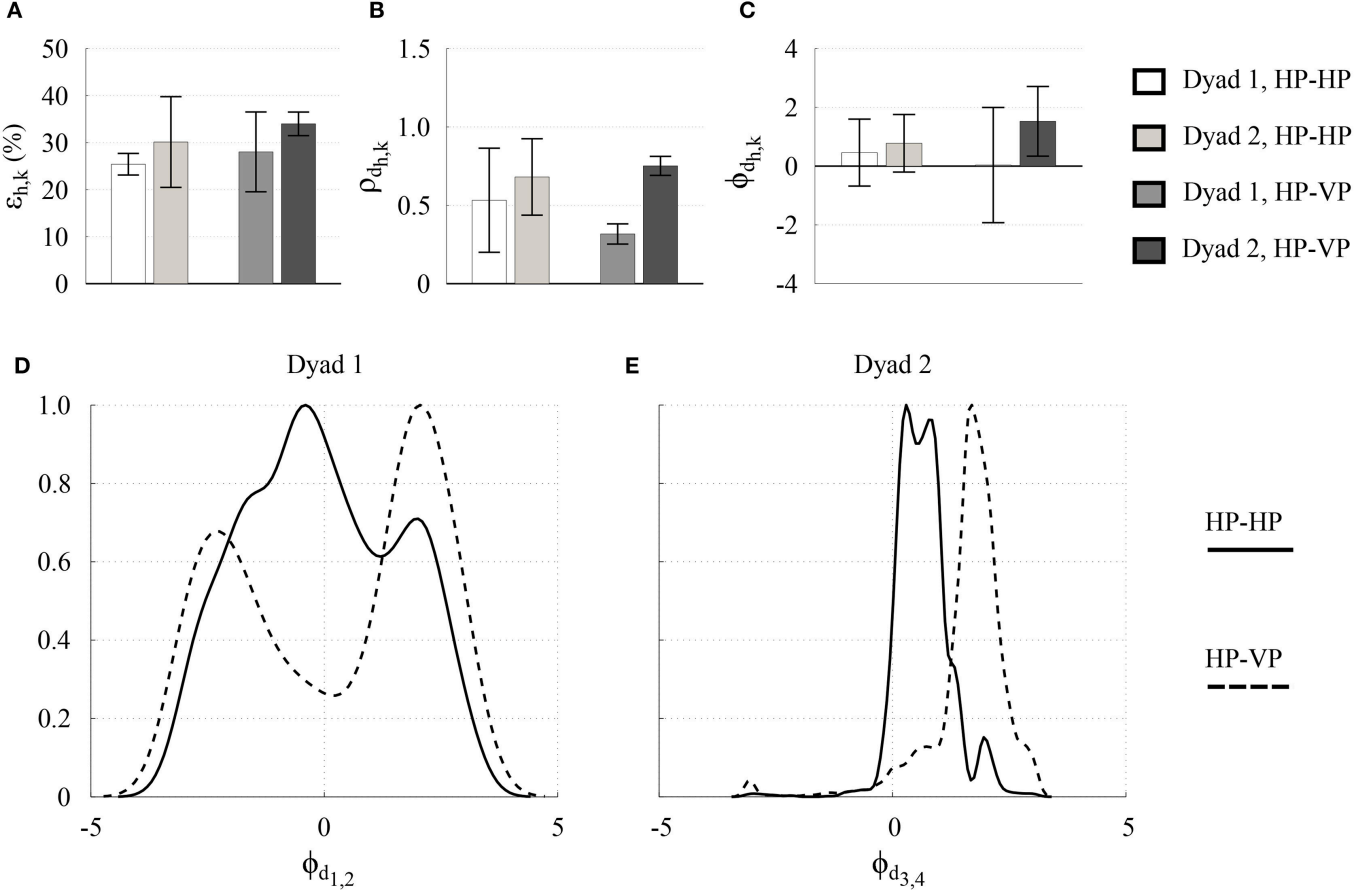
Experimental results in the *Dyadic interaction* experiments. RMS of the normalized position error ϵ_*h,k*_ **(A)**, dyadic synchronization indices ρ_*d*_*h,k*__ **(B)**, and relative phase ϕ_*d*_*h,k*__ between the two participants **(C)** are shown for each pair (Dyad 1 and Dyad 2), where different scales of gray refer to different pairs and players. The height of each bar represents the mean value averaged over the 3 trials for each pair, whereas the black error bar represents its averaged standard deviation. The PDF of the relative phase ϕ_*d*_*h,k*__ between the two participants of Dyad 1 **(D)** and Dyad 2 **(E)** are shown for the first trial of each pair, where the black solid line refers to HP-HP interaction, and the black dashed line refers to HP-VP interaction.

Interestingly, the relationships between the metrics obtained for the two dyads in HP-HP interaction are replicated when substituting one of the two human players in each pair with a virtual agent (Figure 5). This seems to confirm that the VP, as designed in Alderisio et al. (2016a) and implemented in our novel software set-up, is able to interact in a human-like fashion with the other player, becoming a kinematic avatar of the person it is substituting in the game (Zhai et al., 2016). In particular, the RMS of the normalized position error ϵ_1,2_ obtained in Dyad 1 is lower than ϵ_3,4_ obtained in Dyad 2 (Figure 5A), and the same applies to the dyadic synchronization indices ρ_*d*_1,2__ and ρ_*d*_3,4__ (Figure 5B), and for the relative phase ϕ_*d*_1,2__ and ϕ_*d*_3,4__ (Figure 5C). Notably, for both dyads, the probability density function (PDF) of the relative phase obtained for the two players in HP-VP interaction resembles that obtained in HP-HP interaction (Figures 5D–E). Indeed, the PDFs related to Dyad 1 are broader ad centered around 0, whereas those related to Dyad 2 are tighter and shifted on the right.

These experiments were carried out in the second session (see Section 1.2 of Supplementary Material for more details on how to perform dyadic interaction trials via Chronos and replace one of the human players with a VP).

#### 3.3.3. Group interaction experiments

Two different groups of 4 (the same as *Solo experiments* and *Dyadic interaction*) and 5 other participants were separately tested, respectively named Group 1 and Group 2. Participants in each group were asked to synchronize their motion with that of the circles shown on their respective computer screen, representing the movements of the other agents topologically connected with them. However, players had no global information of the topology of their interactions.

##### 3.3.3.1. Mixed HP-VP network

**Figure 6:**
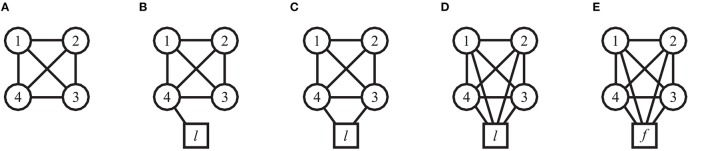
Topology of connections among participants in the *Group interaction* experiments—Group 1. Circles refer to human participants, while the square refers to the virtual player (*l*: leader mode, *f* : follower mode). **(A)** Undirected all-to-all interaction structure (each player sees the motion of all the others), with the addition of undirected links between a VP and 1, 2, or 4 HPs (**B–E**, respectively) are shown. The virtual player acts as a leader in topologies **(B–D)** and as a follower in topology **(E)**.

Four participants (Group 1) were involved in this session. Firstly, 3 trials of 30 s each were performed where all participants saw on their respective screens traces of the objects moved by all the others (all-to-all configuration, Figure 6A). Secondly, a VP (modeled by HKB equation and adaptive control) fed with the sinusoidal motion of a different player was introduced in the network; participants were told that a fifth human player was interacting with them. The virtual agent was first connected in *leader mode* to either 1, 2, or 4 HPs (Figures 6B–D, respectively), and then in *follower mode* to all of them (Figure 6E). For each topology including the virtual player, once again 3 trials of duration 30 s were performed.

**Figure 7:**
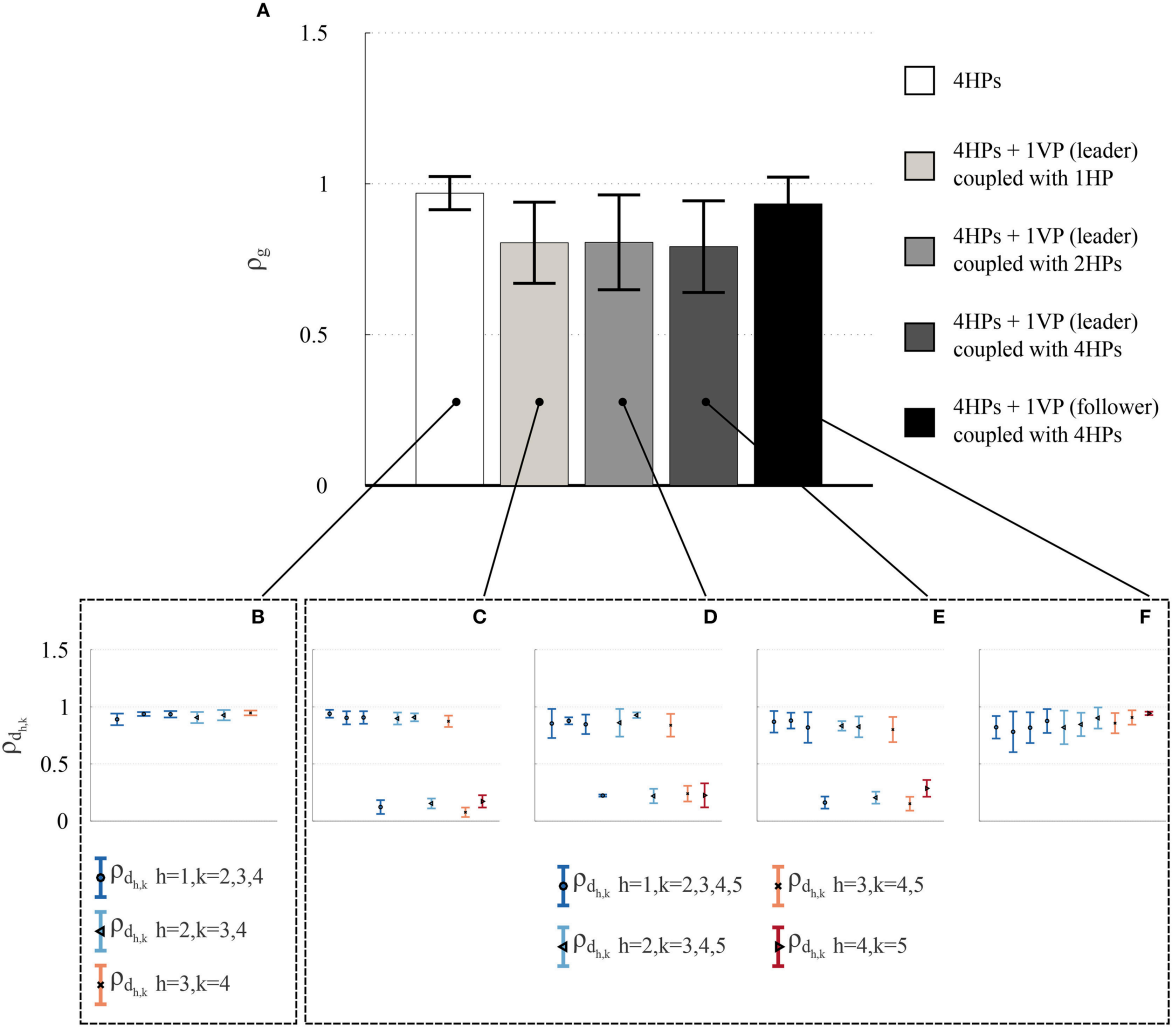
Experimental results in the *Group interaction* experiments—Group 1. The group synchronization indices obtained for the players in the five different topologies of Figure 6 are shown, with different scales of gray representing different topologies **(A)**. The height of each bar represents the mean value over time of the group synchronization index ρ_*g*_(*t*), averaged over the 3 trials for each topology, whereas the black error bar represents its averaged standard deviation. The corresponding dyadic synchronization indices ρ_*d*_*h,k*__ obtained for all the pairs of players in the topologies of Figures 6A–E are respectively shown in **(B–F)**. Different symbols and colors refer to mean and standard deviation averaged over the 3 trials performed for each topology, respectively. As ρ_*d*_*h,k*__ are symmetric by definition, only half of them are depicted.

It is possible to appreciate that the highest value of group synchronization observed experimentally is obtained in the HP network, while lower values are obtained when introducing a VP as leader. However, the group synchronization index ρ_*g*_ increases again when a VP is introduced as follower (Figure 7A). These results are confirmed by the dyadic synchronization indices ρ_*d*_*h,k*__ respectively obtained in the five topologies of interest (Figures 7B–F). For each pair of human players, high values (Figure 7B) are observed for the topology shown in Figure 6A. On the other hand, when a virtual leader is introduced in the interaction (topologies shown in Figures 6B–D) the lowest values of dyadic synchronization are obtained for each human player in correspondence to the VP (player 5 in Figures 7C–E). Finally, when the VP acts as a follower (topology shown in Figure 6E), the highest values of dyadic synchronization indexes ρ_*d*_*h,k*__ for each human player are observed in correspondence to the VP (player 5 in Figure 7F). For more details see Tables S1, S2.

These experiments were carried out in the second session (see Section 1.3 of Supplementary Material for more details on how to perform group interaction trials via Chronos and deploy virtual agents within the human ensemble).

##### 3.3.3.2. HP network

**Figure 8:**
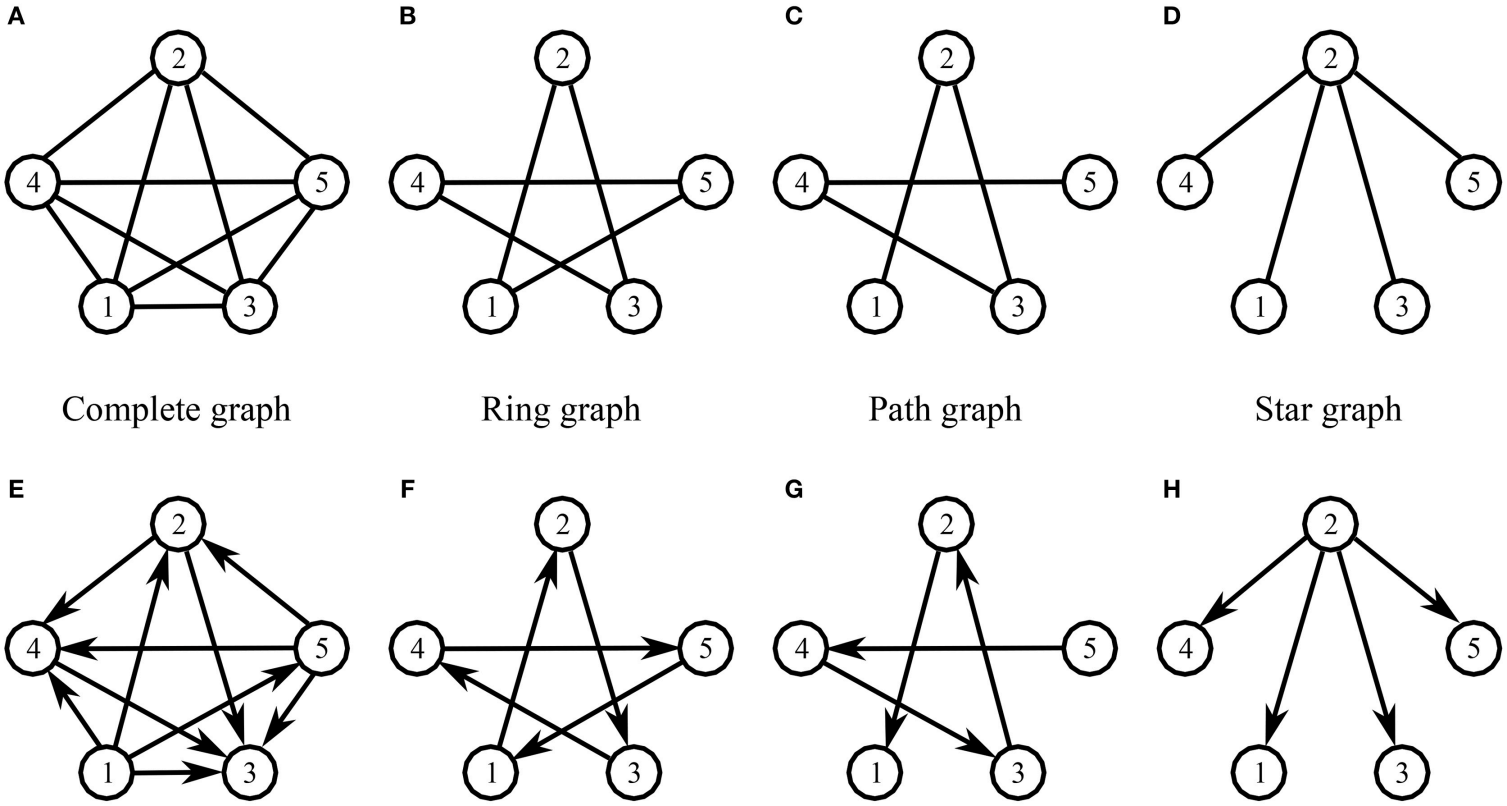
Topology of connections among participants in the *Group interaction* experiments—Group 2. **(A–D)** represent undirected complete, ring, path and star graph, respectively. **(E–H)** represent the respective directed versions. Edges without arrows represent *undirected* connections (if participants *i* sees the motion of participant *j*, then also participant *j* sees the motion of participant *i*), whereas in the *directed* case, an edge going out of node *i* and coming in node *j* (the direction of the edge is given by its corresponding arrow) is representative of the fact that participant *j* sees the motion of participant *i*.

Five participants (Group 2) were involved in this session. Eight different topologies of interactions were implemented among them (Figure 8): undirected complete (Figure 8A), ring (Figure 8B), path (Figure 8C), and Star graph (Figure 8D), and their respective directed version (Figures 8E–H). As for the undirected topologies:

- Complete graph: each participant could see the movements of all the others.
- Ring graph: each participant could see the movement of only two other players, called *neighbors*.
- Path graph: similar to the Ring graph configuration, but two participants (players 1 and 5), defined as *external* participants, could see the movements of only one *neighbor* (respectively players 2 and 4), and as a consequence were not connected to each other.
- Star graph: one participant defined as *central* (player 2) could see the movements of all the others, defined as *peripheral*, who in turn could see the movements of only the *central* player.

For each topology, 6 trials of duration 30 s were performed.

**Figure 9:**
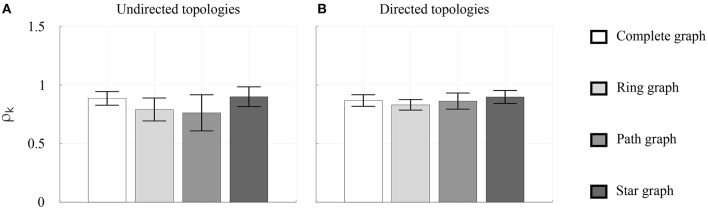
Individual synchronization indices in the *Group interaction* experiments—Group 2. Different scales of gray refer to different topologies. The height of each bar represents the mean value, over the total number of participants, of the individual synchronization index ρ_*k*_ averaged over all the trials, whereas the black error bar represents its standard deviation. The coordination levels are shown in **(A)** for the undirected topologies and in **(B)** for the directed topologies.

The values of the individual synchronization indices ρ_*k*_ of the participants were first averaged over the total number of trials for each *k*th player and for each topology (both in the undirected and in the directed case), and then underwent a one-way ANOVA with repeated measures. Their mean value and standard deviation over the total number of participants are represented for each topology in Figure 9. In the undirected case, the ANOVA performed with Greenhouse–Geisser correction revealed a statistically significant effect of the topology [*F*_(1.201, 4.805)_ = 8.859, *p* < 0.05, η^2^ = 0.689], suggesting an advantage of Complete graph and Star graph (Bonferroni *post-hoc* test, *p* < 0.05). Albeit preliminary, this result seems to confirm independently the observations reported in Alderisio et al. (2016c) showing that undirected interaction patterns among participants affect their coordination level. Also, for all the topologies, higher mean values and lower standard deviations of group synchronization index are observed in the directed case (with the only exception of the former in the Complete graph). For more details see Table S3.

**Figure 10:**
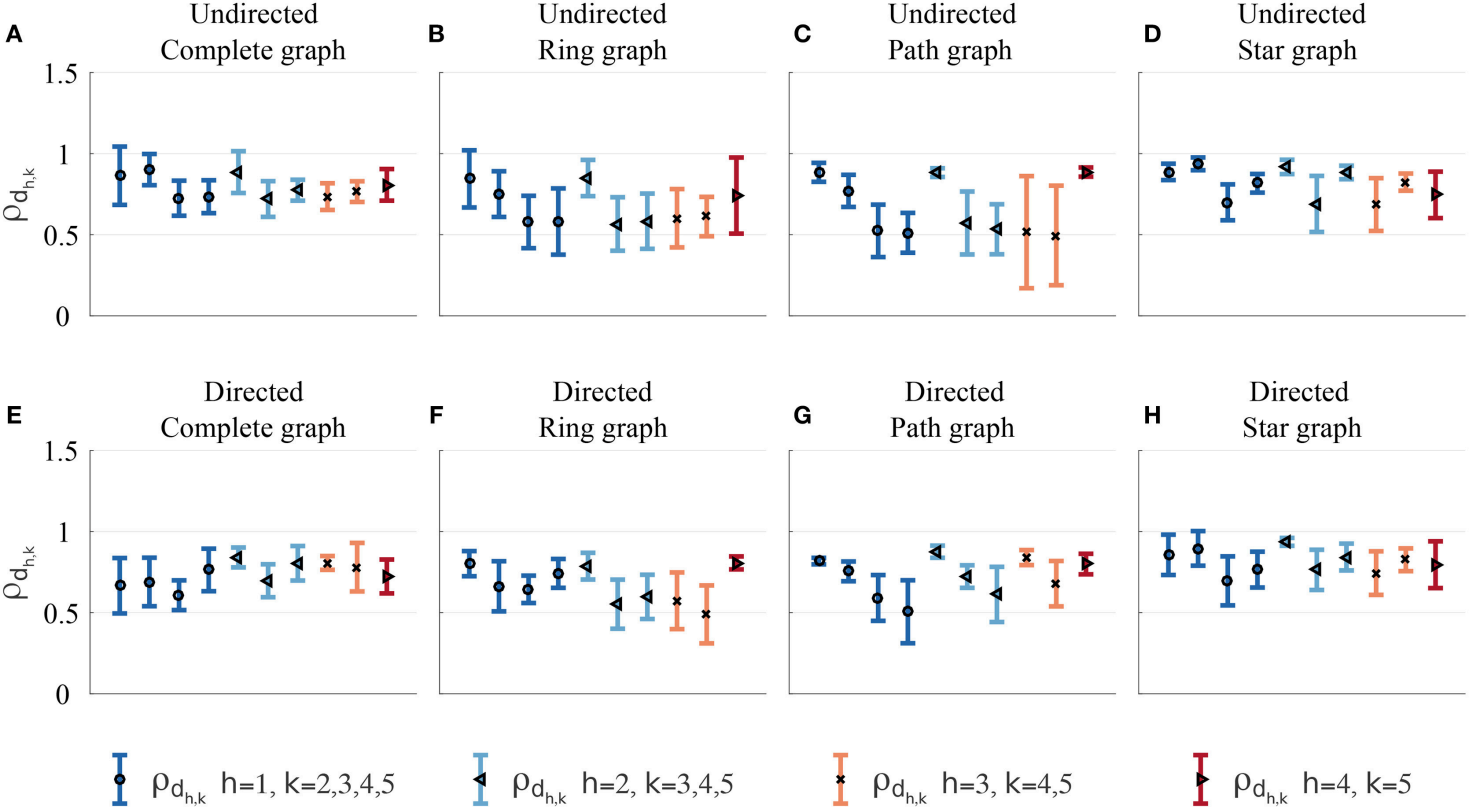
Dyadic synchronization index in the *Group interaction* experiments—Group 2. Different symbols and colors refer to pairs related to different players. Mean (symbol) and standard deviation (error bar) over the total number of trials of the dyadic synchronization indices ρ_*d*_*h,k*__ = ρ_*d*_*k,h*__ in the undirected [**(A)** complete, **(B)** ring, **(C)** path, and **(D)** star graph] and in the respective directed topologies **(E–H)** are shown. As ρ_*d*_*h,k*__ are symmetric by definition, only half of them are depicted.

As expected for both undirected and directed topologies, in most cases (83% for undirected and 91% for directed topologies) the highest mean values of dyadic synchronizations over the total number of trials are observed within topologically connected participants (Figure 10). Statistically, visually paired dyads across both undirected and directed topologies were indeed found to exhibit higher synchronization than non-visually coupled dyads [*t*_(78)_ = −4.544, *p* < 0.01]. For more details see Tables S4, S5.

These experiments were performed in the third session (see Section 1.3 of Supplementary Material for more details on how to perform group interaction trials via Chronos and set different interaction patterns among participants, and Figure S12 for an example of trajectories recorded in a group interaction trial performed by a human ensemble).

## 4. Discussion

In this work we presented an *ad hoc* novel computer-based set-up for investigating human coordination, both in dyads and in groups, and showed preliminary results on coordination in human ensembles in order to validate its effectiveness. The proposed set-up allows to remove the effects of social interactions among the players and to implement different structures of interconnections. In addition, it allows to deploy virtual agents in the group, thus opening the possibility of further investigating the mechanisms that underly human group coordination through an extension of the human dynamic clamp to multiplayer scenarios (Dumas et al., 2014).

We envisage that the computer set-up presented in this paper can be used in Social Psychology to elucidate what the effects of social interactions are in dyadic or group movement coordination. Indeed, joint action tasks might first be performed while allowing participants to share direct visual and auditory coupling (participants directly look at each other instead of the screen of their personal computers, and do not wear headphones so that they know who they are interacting with), and then while removing them (or vice versa). Moreover, since some of the players can be replaced with one or more virtual agents, our computer technology can also be exploited for the development of artificial agents able to merge and interact within a group of humans (Boucenna et al., 2016; Iqbal et al., 2016), both for recreational (Alac et al., 2011) and rehabilitation purposes (Zhai et al., 2015; Bono et al., 2016; Słowiński et al., 2017).

In order to illustrate the features and capabilities of Chronos, we applied the platform to some representative scenarios. Specifically, we validated the use of a virtual player as designed in Alderisio et al. (2016a) in a dyadic interaction task. We found that the behavior exhibited in terms of the metrics used in Section 3.2 by each dyad was the same for both HP-HP and HP-VP interaction. This suggests that the human players involved in our experiments did not change the way of interacting with their partner according to the nature of the latter. In particular, we observed that if a human participant to whom a follower role was assigned in duo interaction ended up leading her/his human partner (in spite of the instruction given), s/he did so also when interacting with a virtual leader (Dyad 1). On the other hand, if a human leader was successfully leading her/his human partner, s/he did so also in the interaction with a virtual follower (Dyad 2). Despite being interesting, such preliminary results are specific for the trials we performed here to validate Chronos, hence they call for more experiments in order to be confirmed and extended.

Moreover, we illustrated the possibility of implementing different interaction patterns in larger ensembles. We observed that, only in the case of undirected topologies, coordination levels in a human ensemble are affected by the specific structure of interconnections among group members when any form of direct visual, auditory or social interaction is removed, a result found also in Alderisio et al. (2016c) yet in the presence of visual and social cues. This leads to open questions on what topology has to be implemented in order to enhance synchronization in the group, what the effects of removing some connections are, and whether the presence of social interaction further increases coordination.

Also, we validated the deployment of virtual agents in a group, and observed that they can decrease coordination levels when acting as leaders. Higher values of group and dyadic synchronization indices were instead observed either when no virtual player was interacting within the human ensemble, or when it was following the motion of all the subjects. These results only suggest that virtual players can be used to vary the level of coordination in a human group, although further work is required to better understand this effect and its implications.

Despite these results being promising, our experiments were presented in this methodological paper mainly to show the features of the computer-based set-up we propose. Rather than being exhaustive, they only illustrate the capabilities of Chronos and the analysis that can be carried out, hence we leave to future publications a more thorough experimental confirmation of the preliminary findings reported here for illustrative purposes.

Some further extensions to our work include the possibility of implementing time-varying topologies to study the effects of dynamically adding/removing connections among interacting participants (Cardillo et al., 2014), and enabling the administrator to provide the players with social cues in real time, based on the quality of their performance (i.e., as measured by the group synchronization index). In addition, it is possible to implement new mathematical models (Snapp-Childs et al., 2011; Zhai et al., 2016) for the VP to perform as joint improviser with other virtual or human agents. Finally, we are exploring the possibility of extending Chronos over the Internet, where it is also necessary to deal with network latency issues.

## Author contributions

Conceived and designed the experiments: FA, GF, MdB. Performed the experiments: FA, ML, GF. Analyzed the data: FA, GF. Contributed analysis tools: FA, GF. Developed the software: ML. Wrote the paper: FA, GF, MdB.

### Conflict of interest statement

The authors declare that the research was conducted in the absence of any commercial or financial relationships that could be construed as a potential conflict of interest.
