# Supplementary material for: A Novel Computer-Based Set-Up to Study Movement Coordination in Human Ensembles
Source: Front Psychol. 2017 Jun 9;8:967. doi: 10.3389/fpsyg.2017.00967 (PMC5465282; doi:10.3389/fpsyg.2017.00967)
Supplement: Supplementary file 1 [file Presentation1.PDF]

frontiers

Supplementary Material: A novel computer-based set-up to study movement coordination in human ensembles

Francesco Alderisio†, Maria Lombardi†, Gianfranco Fiore and Mario di Bernardo*

*Correspondence:
Author Name: Mario di Bernardo
m.dibernardo@bristol.ac.uk

†*These authors have contributed equally as first authors*

1 HOW TO USE CHRONOS

Here, we briefly explain how to use Chronos, our proposed computer-based set-up. For further details and to download the software, follow the link `https://dibernardogroup.github.io/Chronos`.

The hardware equipment necessary for its use consists of:

- *Low-cost motion sensors*. Each player waves her/his index finger over a Leap Motion controller (Leap Motion, Inc.) which captures its movements over time; alternatively, a mouse or trackpad can be used.
- *Personal Computers*. Each motion sensor is connected to a PC, such that the recorded position trajectory can be stored after any trial. Participants are able to see their motion and that of the others they are possibly interacting with on their respective computer screens, by means of moving color-coded circles. One additional computer is needed to run the server and a GUI that allows the administrator to set the experimental parameters and the desired visual interaction patterns.
- *Headphone sets*. Each player wears headphones through which white noise is transmitted to eliminate possible auditory couplings with the others.
- *Router*. It provides Wi-Fi signal in order to allow clients (administrator and players) and server to be logged onto the same wireless local area network (WLAN) through TCP/IP protocol.

For the sake of simplicity, server and administrator modules are run on the same machine, so that they share the same IP address. Before carrying out any experiments, the administrator needs to follow the preliminary steps listed below (only once):

1. turn on the wireless router and connect her/his machine to it;
2. import the motor signature database on her/his machine.

For each trial, human players can select the input device (mouse or Leap Motion) and choose to quit the trial before its end by pressing the *q* key on their keyboard.

1.1 Solo experiments

The administrator:

1. runs the server module on a terminal window;
2. runs the database (MySql server) through *MySql Workbench*;
3. runs the administrator module on a different terminal window, and selects *Add new Signature* (Supplementary Figure S2);
4. at the end of the trial, enters the HP's required details and the kind of motion performed (sinusoidal or free) so that her/his position σ and velocity $\dot{\sigma}$ trajectories can be appropriately stored in the database (Supplementary Figure S3).

The HP performs the experiment.

1.2 Dyadic interaction

Two (or three) machines are necessary to carry out HP-VP (or HP-HP) trials.

- For HP-VP trials, the administrator:
  1. runs the server module on a terminal window of a machine;
  2. runs the database (MySql server) through *MySql Workbench*;
  3. runs the administrator module on a different terminal window and, after selecting *Set dyadic interaction* (Supplementary Figure S2), selects *Human Player - Virtual Player*;
  4. selects trial duration, roles for the interaction (leader or follower) for both HP and VP (Supplementary Figure S4), and inner dynamics, control signal and motor signature (extracted from the database) for the VP – if no parameters are specified, the default values shown in the interface are used.

  Note that motor signatures are selected by choosing them among those stored in the database of all signals acquired during previous *Solo experiments*, thus allowing the VP to exhibit the desired kinematic features of one of the HPs whose signatures have been stored during previous experimental runs. Signatures are indexed in the database through a string identifying the players for which they were stored.
- For HP-HP trials, the administrator:
  1. runs the server module on a terminal window of a machine;
  2. runs the administrator module on a different terminal window and, after selecting *Set dyadic interaction* (Supplementary Figure S2), selects *Human Player - Human Player*;
  3. selects the roles for the interaction (leader, follower or joint improviser) for both HPs (Supplementary Figure S5).

For both types of trials, each HP:

1. connects her/his machine to the WLAN provided by the router;
2. runs her/his client module on a terminal window of her/his machine and, after entering the server's IP address, selects *Dyadic interaction* (Supplementary Figure S6) and enters an integer index (1 or 2, respectively) uniquely identifying herself/himself;
3. performs the experiment (her/his role chosen for the interaction by the administrator is shown in the gaming screen).

An example of gaming screen shown to the HP in the case of *Dyadic interaction* experiments (Leader-Follower condition) is shown in Supplementary Figure S7.

1.3 Group interaction

In the current version of the hardware/software platform, the total number of participants (HPs and/or VPs) needs to belong to the range $[2, 7]$. Denoting with N the total number of HPs and with M the total number of VPs, $N + 1$ machines are necessary to carry out these experiments.

- For mixed HP-VP networks, the administrator:
  1. runs the server module on a terminal window of a machine;
  2. runs the database (MySql server) through *MySql Workbench*;
  3. runs the administrator module on a different terminal window and selects *Set network topology* (Supplementary Figure S2);
  4. selects duration of the trial, number of HPs and VPs (between 2 and $N + M$ combined) and sets the network topology (Supplementary Figure S8);
  5. for each VP, selects its mode (leader or follower), inner dynamics and control (if no parameters are specified, default values are used), motor signature (from the database) and an integer index (between 1 and $N + M$) uniquely identifying it in the network (Supplementary Figure S9).
- For HP-HP networks, the administrator:
  1. runs the server module on a terminal window of a machine;
  2. runs the administrator module on a different terminal window and selects *Set network topology*;
  3. selects duration of the trial, number of HPs (between 2 and N) and sets the network topology (Supplementary Figure S8).

When setting the network topology, entering 1 in position (i, j) corresponds to allowing agent i to see the motion of agent j; 0 has to be entered in position (i, i).

For both types of networks, all HPs:

1. connect their machine to the WLAN provided by the router;
2. run their client module on a terminal window of their own personal computer and, after entering the server's IP address, select *Group interaction* (Supplementary Figure S6) and enter an integer index (between 1 and $N + M$ in the case of HP-VP networks, between 1 and N in the case of HP-HP networks) uniquely identifying them in the network;
3. perform the experiment.

An example of the gaming screen in the case of *Group interaction* experiments among 4 players is shown in Supplementary Figure S10.

1.4 Data storage and file names

For each player (HP or VP), all data can be saved on the server's machine by calling a shell script named *saveData* from its terminal at the end of each trial (see link `https://dibernardogroup.github.io/Chronos` for more information). Data is saved as a *.txt* file (motor signatures are stored in the database as well) made up of three (for *Solo experiments*) or two (for all the other cases) columns: the first contains the time instants (in ms) in which data was sampled, the second contains the sampled position (in dm) and the third (only for *Solo experiments*) the respective velocity (in $\frac{dm}{s}$).

In particular, each file is saved as $\texttt{P}N_p\_0N_t\_Z\_\texttt{1d.txt}$, where

- N_p is the total number of players involved in the trial;
- N_t is an integer index identifying the trial;
- Z uniquely identifies the players involved in the trial.

In the case of *Solo experiments*, a further parameter is added to identify the kind of motion (sinusoidal or free) performed by the human player.

For instance:

- In *Solo experiments*, `P1_03_Sample_free_1d.txt` refers to the third trial of a player called `Sample` who performed a free motion in isolation (`sinusoidal` instead of `free` if the motion performed was a sine wave);
- In *Dyadic interaction (Leader-Follower condition)*, `P2_03_L_1d.txt` refers to the third trial of the leader (`F` instead of `L` for the follower);
- In *Dyadic interaction (Joint improvisation condition)*, `P2_03_JI1_1d.txt` refers to the third trial of the player identified with index `1` (`JI2` instead of `JI1` for the other player);
- In *Group interaction*, `P5_02_4_1d.txt` refers to the second trial of the player identified with index `4` in a network of `5` participants.

2 SUPPLEMENTARY TABLES AND FIGURES

2.1 Tables

Table S1. Mean value $\mu(\rho_g)$ and standard deviation $\sigma(\rho_g)$ over time of the group synchronization index in the *Group interaction* experiments, averaged over the 3 trials for each topology – Group 1. This table shows $\mu(\rho_g) \pm \sigma(\rho_g)$ for the 5 topologies of interest.

**Topology**	**All-to-all**
4 HPs	0.9689 ± 0.0551
4 HPs + 1VP (leader) connected to 1 HP	0.8044 ± 0.1346
4 HPs + 1VP (leader) connected to 2 HPs	0.8057 ± 0.1572
4 HPs + 1VP (leader) connected to 4 HPs	0.7915 ± 0.1518
4 HPs + 1VP (follower) connected to 4 HPs	0.9331 ± 0.0887

Table S2. Mean value of the dyadic synchronization indices $\rho_{d_{h,k}}$ over the 3 trials for each of the 5 topologies of interest in the *Group interaction* experiments – Group 1. Player 5 represents the additional VP.

4 HPs – Agents	1	2	3	4	5
1	-	0.89	0.94	0.94	-
2	0.89	-	0.91	0.93	-
3	0.94	0.91	-	0.95	-
4	0.94	0.93	0.95	-	-
5	-	-	-	-	-

4 HPs + 1VP (leader) connected to 1 HP – Agents	1	2	3	4	5
1	-	0.94	0.90	0.91	0.12
2	0.94	-	0.90	0.91	0.15
3	0.90	0.90	-	0.87	0.08
4	0.91	0.91	0.87	-	0.17
5	0.12	0.15	0.08	0.17	-

4 HPs + 1VP (leader) connected to 2 HPs – Agents	1	2	3	4	5
1	-	0.85	0.88	0.85	0.22
2	0.85	-	0.86	0.93	0.22
3	0.88	0.86	-	0.84	0.24
4	0.85	0.93	0.84	-	0.22
5	0.22	0.22	0.24	0.22	-

4 HPs + 1VP (leader) connected to 4 HPs – Agents	1	2	3	4	5
1	-	0.87	0.88	0.82	0.16
2	0.87	-	0.83	0.83	0.20
3	0.88	0.83	-	0.80	0.15
4	0.82	0.83	0.80	-	0.29
5	0.16	0.20	0.15	0.29	-

4 HPs + 1VP (follower) connected to 4 HPs – Agents	1	2	3	4	5
1	-	0.82	0.78	0.82	0.88
2	0.82	-	0.82	0.85	0.90
3	0.78	0.82	-	0.86	0.91
4	0.82	0.85	0.86	-	0.94
5	0.88	0.90	0.91	0.94	-

Table S3. Mean value $\mu(\rho_g)$ and standard deviation $\sigma(\rho_g)$ over time of the group synchronization index in the *Group interaction* experiments, averaged over the total number of trials – Group 2. This table shows $\mu(\rho_g) \pm \sigma(\rho_g)$ for both undirected and directed topologies.

**Topology**	**Undirected**	**Directed**
Complete graph	0.8888 ± 0.1925	0.8694 ± 0.1616
Ring graph	0.8145 ± 0.2203	0.8322 ± 0.1864
Path graph	0.7814 ± 0.2068	0.8643 ± 0.1611
Star graph	0.9028 ± 0.1702	0.9070 ± 0.1451

Table S4. Mean value, over the total number of trials, of the dyadic synchronization indices $\rho_{d_{h,k}}$ in the undirected complete, ring, path and star graph in the *Group interaction* experiments – Group 2. Indices related to players who were coupled to each other in the experiments (there exists an edge between them in the respective underlying topology) are represented in bold.

Complete graph – Agents	1	2	3	4	5
1	-	**0.86**	**0.90**	**0.73**	**0.73**
2	**0.86**	-	**0.89**	**0.72**	**0.77**
3	**0.90**	**0.89**	-	**0.73**	**0.77**
4	**0.73**	**0.72**	**0.73**	-	**0.81**
5	**0.73**	**0.77**	**0.77**	**0.81**	-

Ring graph – Agents	1	2	3	4	5
1	-	**0.84**	0.75	0.58	**0.58**
2	**0.84**	-	**0.85**	0.57	0.58
3	0.75	**0.85**	-	**0.60**	0.61
4	0.58	0.57	**0.60**	-	**0.74**
5	**0.58**	0.58	0.61	**0.74**	-

Path graph – Agents	1	2	3	4	5
1	-	**0.88**	0.77	0.52	0.51
2	**0.88**	-	**0.88**	0.57	0.53
3	0.77	**0.88**	-	**0.52**	0.50
4	0.52	0.57	**0.52**	-	**0.89**
5	0.51	0.53	0.50	**0.89**	-

Star graph – Agents	1	2	3	4	5
1	-	**0.89**	0.94	0.70	0.82
2	**0.89**	-	**0.92**	**0.69**	**0.88**
3	0.94	**0.92**	-	0.69	0.82
4	0.70	**0.69**	0.69	-	0.75
5	0.82	**0.88**	0.82	0.75	-

Table S5. Mean value, over the total number of trials, of the dyadic synchronization indices $\rho_{d_{h,k}}$ in the directed complete, ring, path and star graph in the *Group interaction* experiments – Group 2. Indices related to players who were coupled to each other in the experiments (there exists an edge between them in the respective underlying topology) are represented in bold.

Complete graph – Agents	1	2	3	4	5
1	-	**0.67**	**0.69**	**0.61**	**0.76**
2	**0.67**	-	**0.84**	**0.70**	**0.80**
3	**0.69**	**0.84**	-	**0.81**	**0.78**
4	**0.61**	**0.70**	**0.81**	-	**0.72**
5	**0.76**	**0.80**	**0.78**	**0.72**	-

Ring graph – Agents	1	2	3	4	5
1	-	**0.80**	0.66	0.64	**0.74**
2	**0.80**	-	**0.79**	0.55	0.60
3	0.66	**0.79**	-	**0.57**	0.49
4	0.64	0.55	**0.57**	-	**0.81**
5	**0.74**	0.60	0.49	**0.81**	-

Path graph – Agents	1	2	3	4	5
1	-	**0.82**	0.75	0.59	0.51
2	**0.82**	-	**0.88**	0.72	0.61
3	0.75	**0.88**	-	**0.84**	0.68
4	0.59	0.72	**0.84**	-	**0.80**
5	0.51	0.61	0.68	**0.80**	-

Star graph – Agents	1	2	3	4	5
1	-	**0.86**	0.90	0.70	0.77
2	**0.86**	-	**0.94**	**0.76**	**0.84**
3	0.90	**0.94**	-	0.74	0.83
4	0.70	**0.76**	0.74	-	0.80
5	0.77	**0.84**	0.83	0.80	-

2.2 Figures

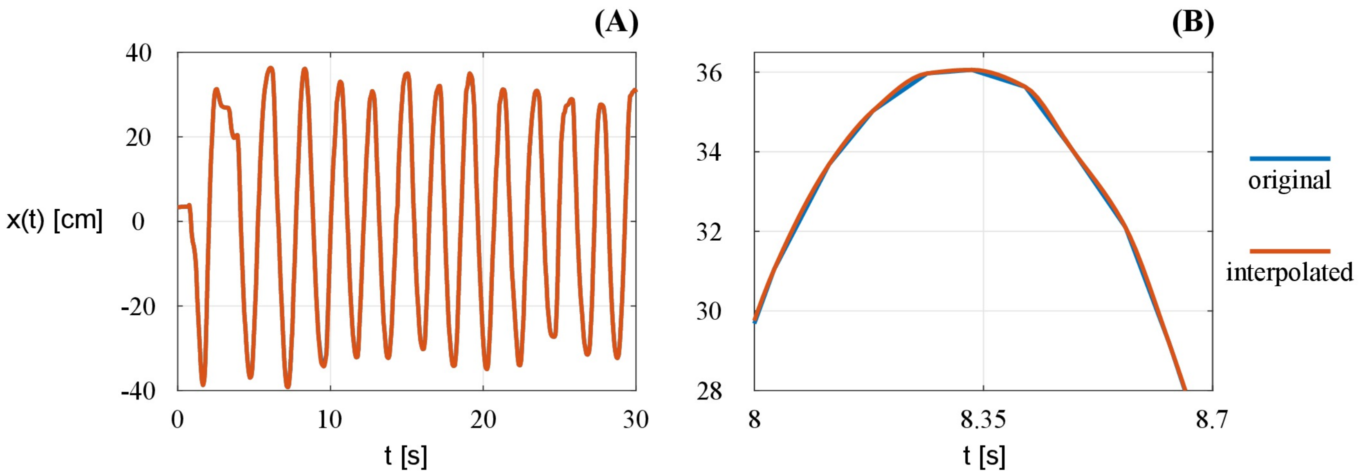

Figure S1. Cubic interpolation for a player's trajectory in *Group interaction* – Group 2. (A) Original (blue line) and interpolated (orange line) position trajectory extracted from a trial of duration $30s$, and respective zoom (B), are shown. Data was originally stored with a frequency rate of $10Hz$, and then underwent cubic interpolation ($100Hz$).

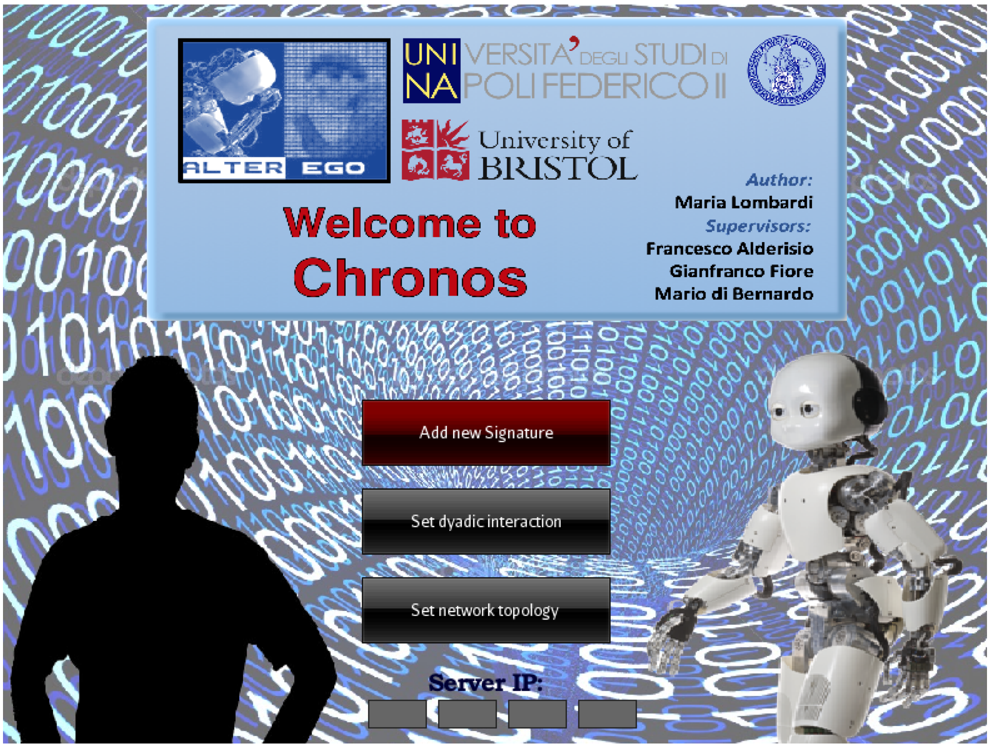

Figure S2. Administrator interface. The administrator selects *Add new Signature* for *Solo experiments*, *Set dyadic interaction* for *Dyadic interaction* experiments, and *Set network topology* for *Group interaction* experiments. If server and administrator modules are run on two different machines, it is necessary to enter the IP address of the former in the appropriate text boxes.

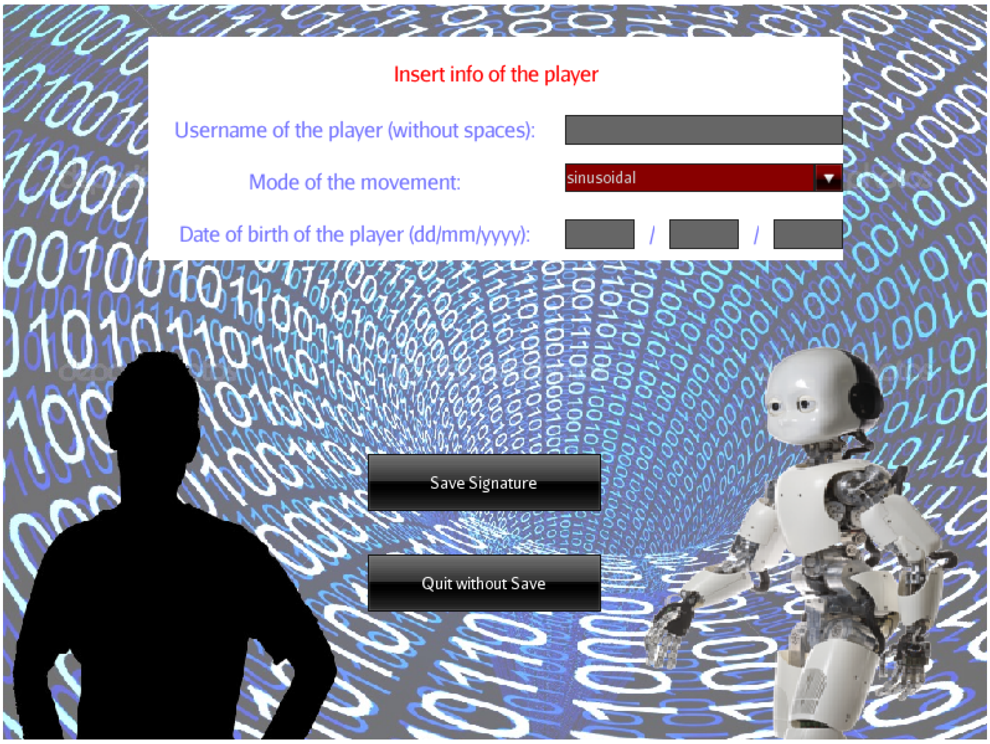

Figure S3. Motor signature storage. The administrator enters data to appropriately store the motor signature of a human player, recorded in *Solo experiments*, in the database.

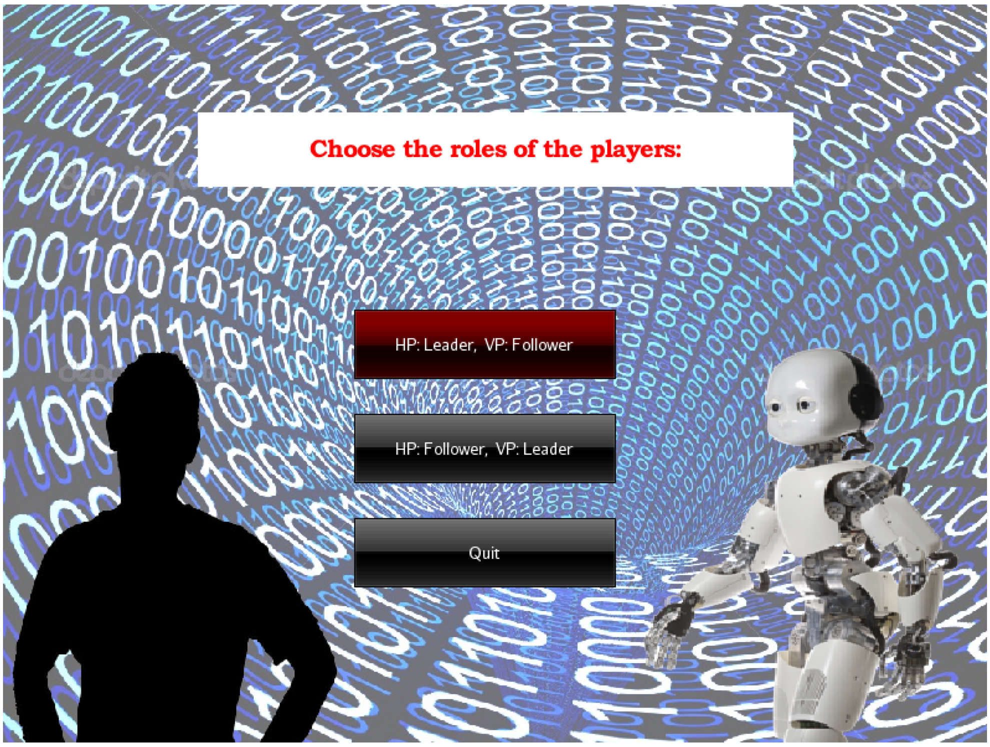

Figure S4. Roles of human and virtual player in *Dyadic interaction*. The administrator sets the roles of HP and VP.

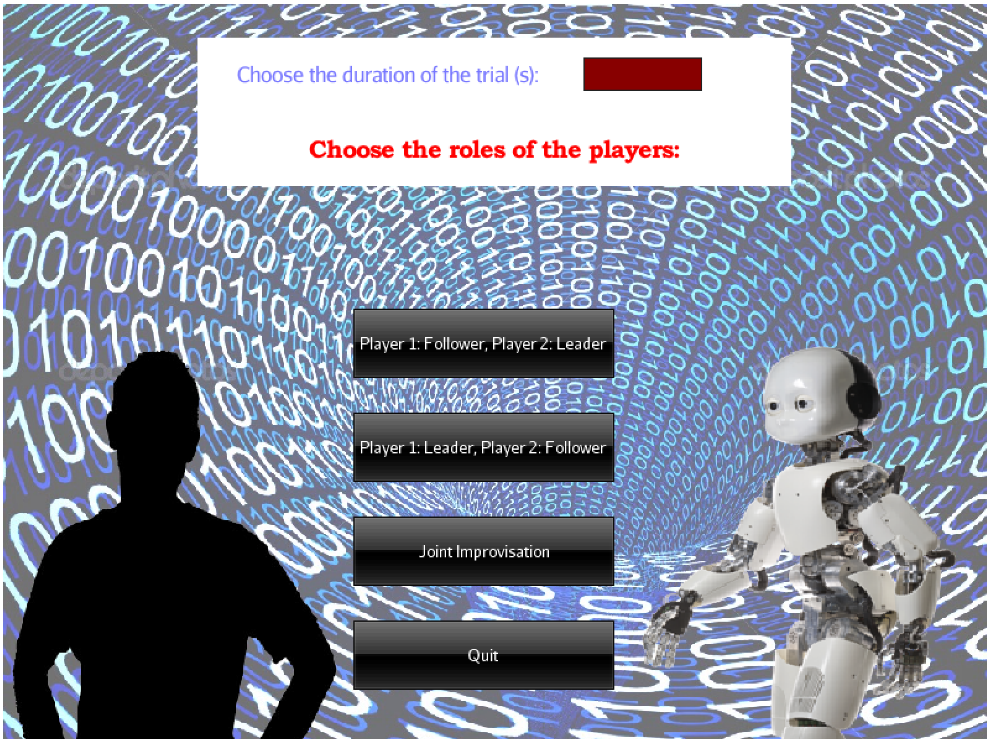

Figure S5. Roles of human players in *Dyadic interaction*. The administrator sets the roles of the two HPs.

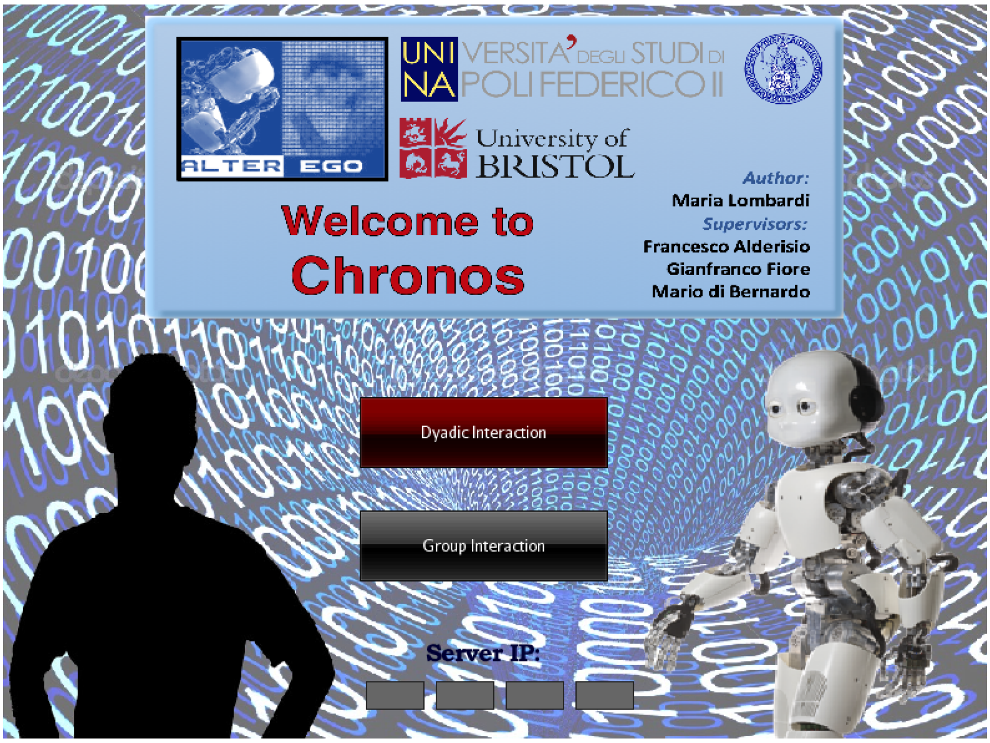

Figure S6. Screen for each human participant to choose the kind of trial to be performed. The player needs to enter the IP address of the server in the appropriate text boxes.

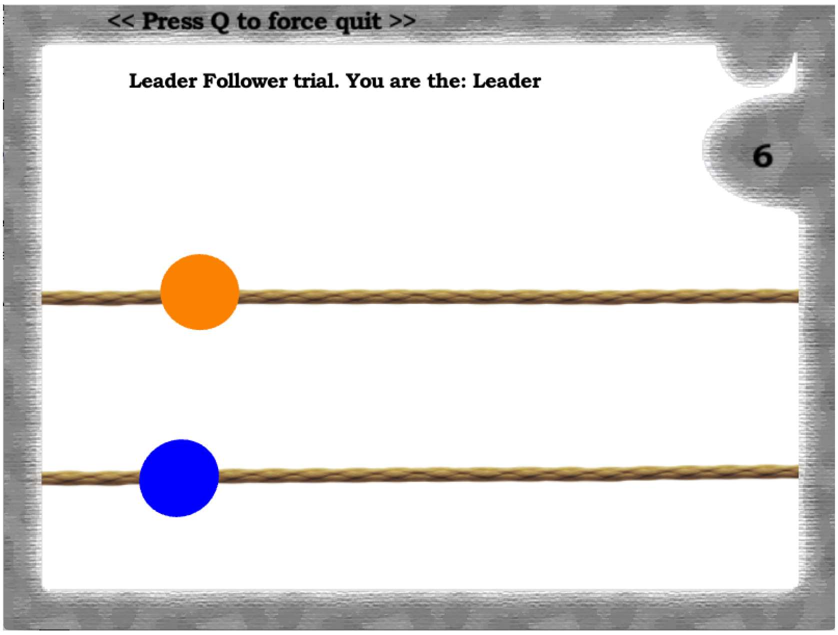

Figure S7. Gaming screen for human participants in *Dyadic interaction* experiments (Leader-Follower condition). Each participant sees her/his own motion represented by a blue circle, and that of the others s/he is interacting with represented by an orange circle. In this case, the gaming screen of the Leader is shown. Top-right: countdown timer (in s).

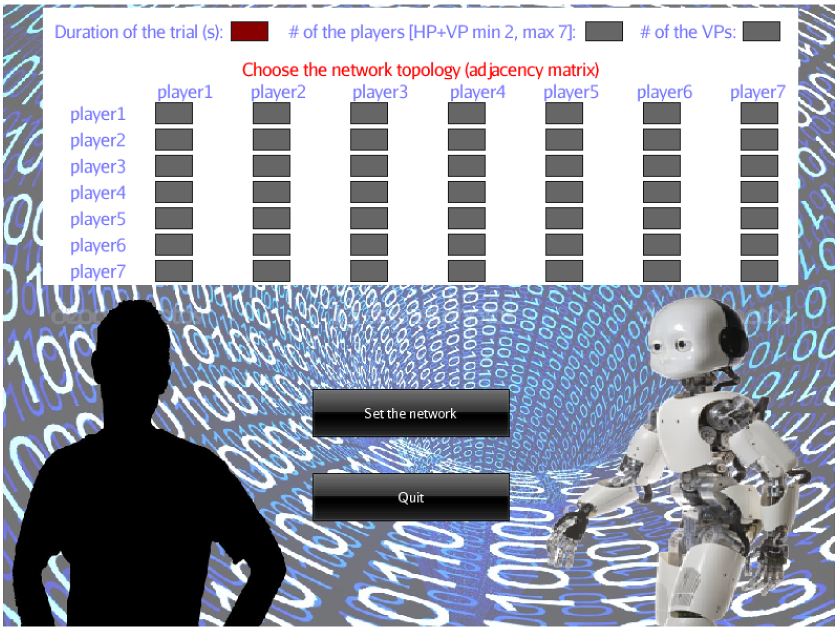

Figure S8. Screen for the administrator to select duration of the trial, number of players and set the network topology. When setting the network topology, entering 1 in position (i, j) corresponds to allowing agent i to see the motion of agent j; 0 has to be entered in position (i, i).

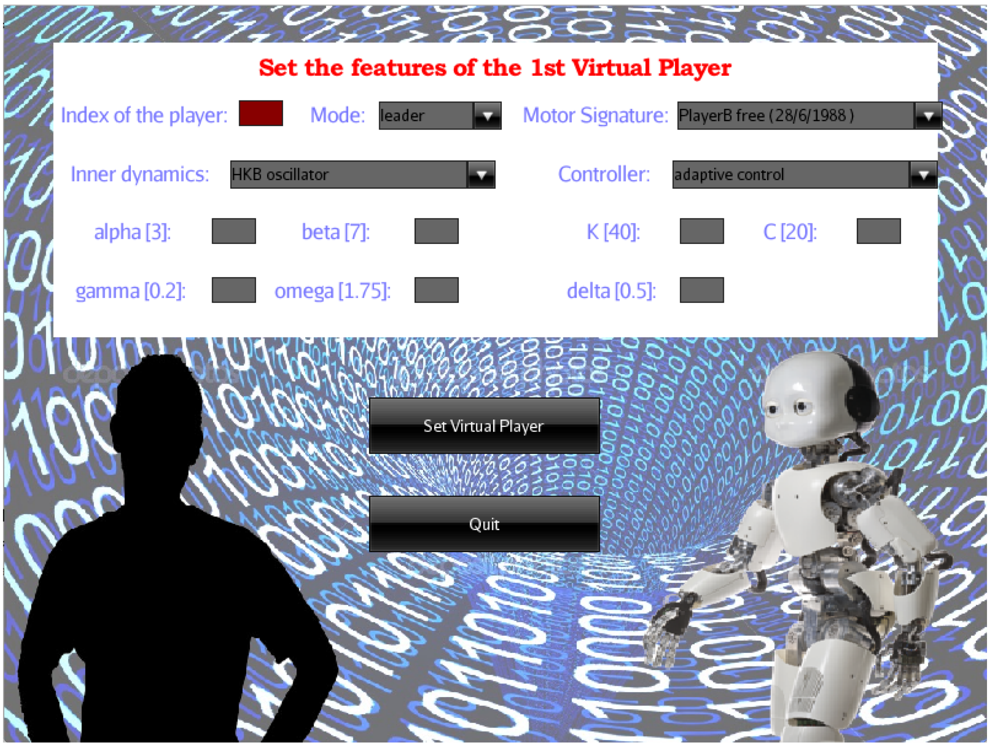

Figure S9. Screen for the administrator to set the VP in *Group interaction* experiments. For each VP, the administrator selects its mode (leader or follower), inner dynamics and control (if no parameters are specified, the default values in the brackets are used), motor signature (from the database) and an integer index uniquely identifying the virtual agent in the network.

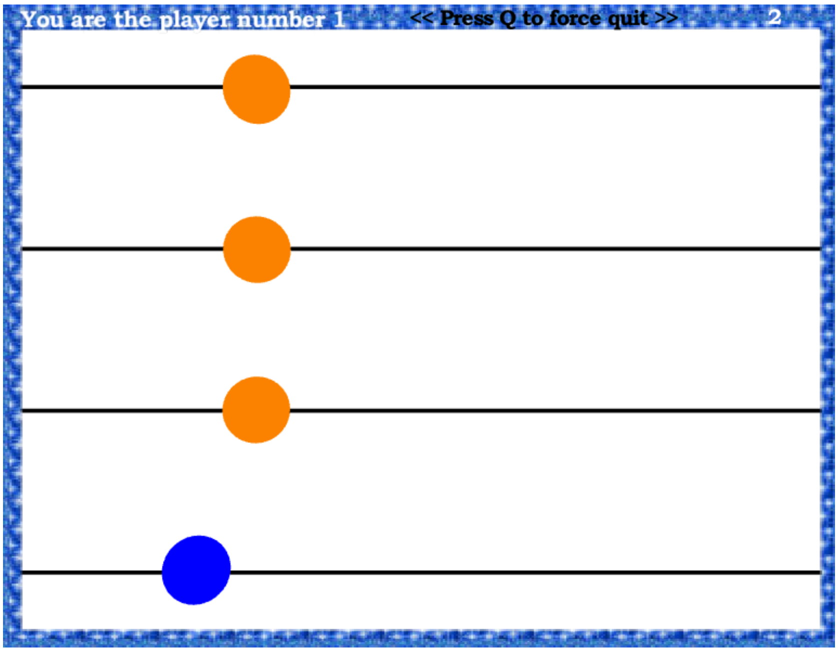

Figure S10. Gaming screen for human participants in *Group interaction* experiments. Each participant sees her/his own motion represented by a blue circle, and those of the others s/he is topologically connected with represented by orange circles, respectively. Top-left: index of the player in the network. Top-right: countdown timer (in s).

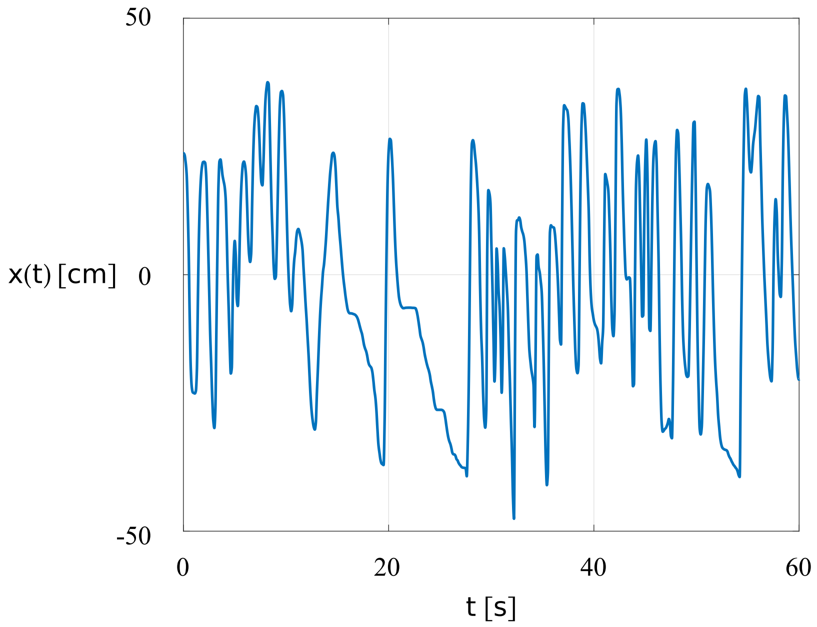

Figure S11. Example of position trajectory $x(t)$ recorded during a *Solo* trial. The instruction given to the player was to "produce an interesting non-periodic motion while oscillating the index finger back and forth over the leap motion".

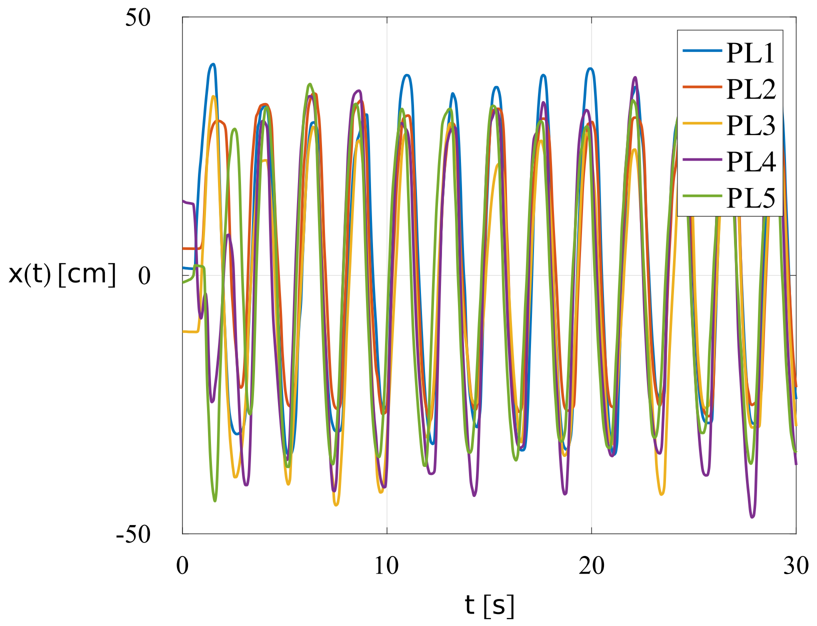

Figure S12. Example of position trajectories $x(t)$ recorded during a *Group interaction* trial in a human ensemble. The instruction given to the players was to "synchronize their motion with that of the circles shown on their screen". Each color refers to a different player, labelled as *PL*.
